# Indolocarbazoles with Sterically Unrestricted Electron-Accepting Anchors Showcasing Aggregation-Induced Thermally Activated Delayed Mechanoluminescence for Host-Free Organic Light-Emitting Diodes

**DOI:** 10.3390/molecules28165999

**Published:** 2023-08-11

**Authors:** Malek Mahmoudi, Ervinas Urbonas, Dmytro Volyniuk, Dalius Gudeika, Kestutis Dabrovolskas, Jurate Simokaitiene, Asta Dabuliene, Rasa Keruckiene, Karolis Leitonas, Matas Guzauskas, Levani Skhirtladze, Marija Stanitska, Juozas Vidas Grazulevicius

**Affiliations:** Department of Polymer Chemistry and Technology, Kaunas University of Technology, K. Barsausko g. 59, 51423 Kaunas, Lithuaniakestutis.dabrovolskas@ktu.lt (K.D.); jurate.simokaitiene@ktu.lt (J.S.);

**Keywords:** indolocarbazole, benzonitrile, mechanochromic luminescence, aggregation-induced emission enhancement, thermally activated delayed fluorescence, organic light-emitting diode

## Abstract

We investigated the effects of sterically nonrestricted electron-accepting substituents of three isomeric indolocarbazole derivatives on their aggregation-induced emission enhancement, mechanochromic luminescence and thermally activated delayed fluorescence. The compounds are potentially efficient emitters for host-free organic light-emitting diodes. The films of indolocarbazole derivatives exhibit emissions with wavelengths of fluorescence intensity maxima from 483 to 500 nm and photoluminescence quantum yields from 31 to 58%. The ionization potentials of the solid samples, measured by photoelectron emission spectrometry, are in the narrow range of 5.78–5.99 eV. The electron affinities of the solid samples are in the range of 2.99–3.19 eV. The layers of the derivatives show diverse charge-transporting properties with maximum hole mobility reaching 10^−4^ cm^2^/Vs at high electric fields. An organic light-emitting diode with a light-emitting layer of neat compound shows a turn-on voltage of 4.1 V, a maximum brightness of 24,800 cd/m^2^, a maximum current efficiency of 12.5 cd/A and an external quantum efficiency of ca. 4.8%. When the compounds are used as hosts, green electroluminescent devices with an external quantum efficiency of ca. 11% are obtained. The linking topology of the isomeric derivatives of indolo[2,3-a]carbazole and indolo[3,2-b]carbazole and the electron-accepting anchors influences their properties differently, such as aggregation-induced emission enhancement, mechanochromic luminescence, thermally activated delayed fluorescence, charge-transporting, and electroluminescent properties. The derivative indolo[3,2-b]carbazole displays good light-emitting properties, while the derivatives of indolo[2,3-a]carbazole show good hosting properties, which make them useful for application in electroluminescent devices.

## 1. Introduction

Organic light emitting diodes (OLEDs) are utilized in flat-panel displays and solid-state lighting technologies due to their advantages of flexibility, color tunability, transparency and efficiency [1,2,3,4]. Because of these properties, OLEDs and organic semiconductors are the objects of great commercial interest. Many organic semiconductors show very different light-emitting properties in dilute and concentrated solutions. Most of the aromatic hydrocarbons emit brightly in dilute solutions but show weak emissions at high concentrations [5]. The main reason for the quenching process is the formation of aggregates which result in aggregation-caused quenching (ACQ) [6]. The ACQ effect can be a major disadvantage in the fabrication of OLEDs, in which thin solid films of organic semiconductors are used [7]. Since the ACQ effect is disadvantageous with respect to applications or organic emitters, researchers have made numerous efforts to solve this problem. As a result, the concepts of aggregation-induced emission (AIE) and aggregation-induced emission enhancement (AIEE) were introduced by Tang et al. [6,8]. AIE is an intriguing photophysical process in which non-emissive molecules in solutions emit strongly in the aggregate or solid state. AIEE is a similar phenomenon to AIE except that, in this case, compounds show a noticeable emission which becomes stronger with an increase in luminophore concentration [9]. Numerous efforts are continually being made in order to explain the working principle of AIE/AIEE [10]. The most popular AIE/AIEE mechanism at the single molecular level involves the restriction of intramolecular rotation [9,11]. The main idea of this mechanism is that some molecules have phenyl rotors which in dilute solution can dynamically rotate against a stator via the C-C single-bonds. This rotation works as a non-radiative decay pathway for the excited states [12]. Such rotations in aggregates are inhibited due to the physical restrictions from surrounding molecules. In this way, the non-radiative pathway is blocked, leading to the radiative decay pathway becoming dominant. However, some new AIE systems that do not have multiple rotors cannot be fully explained by the restriction of intramolecular rotation. To explain this, the restriction of intramolecular vibrations was introduced [13]. The main idea behind the latter mechanism is that under aggregation, the substantial intramolecular vibrations are restricted and, in this way, non-radiative decay pathways are blocked [14]. The combination of two mechanisms is known as the restriction of the intramolecular motion [15,16]. Nowadays, it is the most popular mechanism that explains AIE in a simple and fundamental way [17]. It is achievable to attain a remarkable photoluminescence quantum yield (PLQY) by solid films through AIE/AIEE, without the requirement of doping [18]. Because of that, it is possible to exploit emitters exhibiting AIE/AIEE in efficient host-free OLEDs [19,20]. The number of AIE/AIEE-active chromophores containing tri- and tetraphenylethylene [21,22], pentaphenylsilole [23], benzophenone [24] and many other moieties have already been reported. However, because of the spin statistics [25], the 5% limit of external quantum efficiency (EQE) cannot be overcome for host-free singlet emission-based OLEDs even with perfect AIE-active fluorescent emitters. In order to overcome this barrier, emitters exhibiting thermally activated delayed fluorescence (TADF) can be used. However, such emitters often require appropriate hosts and a special molecular design for applications in electroluminescent devices [26]. The host materials can affect considerably the efficiency of OLEDs. There are many publications which report on host materials for OLEDs [27,28,29]. In OLEDs, with a working principle based on the TADF mechanism, the emitter is usually molecularly dispersed in some type of host matrix at relatively low concentration to prevent exciton quenching in the emitting layer (EML) [30]. Host materials for OLEDs are required to fulfil several mandatory conditions to maximize the efficiency of the devices [31]. The triplet energy level of the host material has to be higher than that of the emitter. The HOMO and LUMO of a host have to match with the HOMO and LUMO of the charge-transporting and emitting materials; a host has to have a higher energy gap between the HOMO and LUMO than a guest. In addition, the layers of good host materials have to show high morphological and thermal stability and high and balanced electron and hole mobilities. Because of the problems related to guest–host systems, OLEDs with host-free EMLs have attracted a great deal of attention. In this work, we aimed to develop emitters exhibiting both AIEE and TADF for host-free OLEDs. To be efficient, singlet emitters have to exhibit bipolar charge-transporting properties [32]. For this, in this work, one rigid donor indolocarbazole moiety and two trifluoromethyl-substituted benzonitrile acceptor moieties were selected for the design of the emitters. To reach a stable color of electroluminescence under the different voltages, multi-conformer formation has to be prevented for singlet emitters [33]. However, this requirement is in contradiction with the above-mentioned requirements for AIEE emitters (the presence of multiple rotors, i.e., flexible or sterically unrestricted moieties in the molecular structures). In order to resolve that contradiction, sterically unrestricted electron-accepting moieties and the central indolocarbazole unit were linked. In addition, the trifluoromethyl-substituted benzonitrile moieties were chosen for their ability to serve as anchors in solid-state films through hydrogen bonding [34,35].

In this work, we developed host-free OLEDs exploiting the newly synthesized indolocarbazole derivatives with electron-accepting groups having anchoring abilities. The developed emitters exhibit AIEE, TADF and mechanochromic luminescence with variations of the wavelengths of photoluminescence maxima in the narrow range. In order to estimate the applicability in OLEDs, the photophysical electrochemical and photoelectrical properties of the differently acceptor-substituted indolocarbazoles were studied. PLQYs of one compound exceeded 50% in solid-state. An OLED with the emitting layer of this compound showed stable electroluminescence colors under different external voltages and an EQE up to 4.8%. In addition, the hosting properties of compounds were tested in green electroluminescent devices reaching external quantum efficiency of ca. 11%.

## 2. Experimental Section

### 2.1. Materials

Indolo[2,3-*a*]carbazole, indolo[3,2-*b*]carbazole, 2-fluoro-5-(trifluoromethyl)benzonitrile, 4-fluoro-3-(trifluoromethyl)benzonitrile, *N*,*N*′-di(1-naphthyl)-*N*,*N*′-diphenyl-(1,1′-biphenyl)-4,4′-diamine (NPB), 4,4′-cyclohexylidenebis[*N*,*N*-bis(4-methylphenyl)benzenamine] (TAPC), 1,3-bis(9-carbazolyl)benzene (mCP), diphenyl-4-triphenylsilyl-phenylphosphineoxide (TSPO1), 1,4,5,8,9,11-hexaazatriphenylenehexacarbonitrile (HAT-CN) and 2,2′,2″-(1,3,5-benzinetriyl)-tris(1-phenyl-1-H-benzimi-dazole) (TPBi) were purchased from Sigma-Aldrich and LUMTEC and used as received.

### 2.2. Synthesis and Identification

#### 2.2.1. 6,6′-(Indolo[3,2-*b*]carbazole-5,11-diyl)bis(3-(trifluoromethyl)benzonitrile) (**1IndCz**)

The solution of indolo[3,2-*b*]carbazole (0.5 g, 1.95 mmol) in anhydrous DMF (10 mL) was added dropwise into DMF (5 mL) solution containing NaH (0.19 g, 7.8 mmol) and stirred for 1 h under nitrogen atmosphere. Then, a solution of 2-fluoro-5-(trifluoromethyl)benzonitrile (0.81 g, 4.29 mmol) in DMF (5 mL) was added dropwise, and the solution was stirred for 24 h at room temperature. When the reaction was completed, water was added into the reaction mixture and the mixture was extracted with dichloromethane. The solvent was removed under vacuum. The crude product was purified by column chromatography on silica gel (eluent chloroform/*n*-hexane, 1:2). Yellow solid was obtained. FW = 594.52 g/mol, yield: 0.76 g, 66%. ^1^H NMR (400 MHz, DMSO, δ, ppm): 8.54 (m, 2H), 8.42 (s, 1H), 8.39–8.29 (m, 3H), 8.26 (d, *J* = 7.5 Hz, 2H), 8.20 (d, *J* = 7.5 Hz, 2H), 7.47 (t, *J* = 7.7 Hz, 2H), 7.37–7.19 (m, 4H). ^13^C NMR (101 MHz, DMSO, δ, ppm): 141.99, 141.96, 141.37, 141.31, 137.36, 137.34, 137.01, 136.95, 135.67, 135.63, 135.34, 135.30, 127.93, 127.89, 127.65, 127.61, 127.30, 126.74, 126.70, 126.66, 124.79, 123.90, 123.84, 123.78, 122.07, 121.69, 121.66, 121.08, 121.00, 116.79, 116.74, 115.91, 115.86, 110.08, 109.97, 101.49, 101.42. MS (ES^+^, 20 V), *m/z*: 595 ([M + H]^+^). Elemental analysis calcd (%) for C_34_H_16_F_6_N_4_: C, 68.69; H, 2.71; F, 19.17; N, 9.42; Found: C, 68.71; H, 2.76; N, 9.41.

#### 2.2.2. 6,6′-(Indolo[2,3-*a*]carbazole-11,12-diyl)bis(3-(trifluoromethyl)benzonitrile) (**2IndCz**)

**2IndCz** was prepared according to the method similar to that used for the preparation of **1IndCz**, except that indolo[2,3-*a*]carbazole (0.5 g, 1.95 mmol), 2-fluoro-5-(trifluoromethyl)benzonitrile (0.81 g, 4.29 mmol) were used. The crude product was purified by column chromatography on silica gel (eluent chloroform/*n*-hexane, 1:2), and crystallized from the mixture of chloroform and hexane. Yellow crystals were obtained. M.p. 282–283 °C, FW = 594.52 g/mol, yield: 0.85 g, 74%. ^1^H NMR (400 MHz, DMSO, δ, ppm): 8.35–8.29 (m, 4H), 8.01 (d, *J* = 8.2 Hz, 1H), 7.94 (d, *J* = 8.2 Hz, 1H), 7.82 (d, *J* = 8.2 Hz, 2H), 7.66 (s, 1H), 7.39–7.33 (m, 5H), 7.09–7.00 (m, 2H). ^13^C NMR (101 MHz, DMSO, δ, ppm): 142.96, 142.92, 142.42, 142.10, 136.89, 136.51, 135.33, 135.00, 134.83, 134.50, 127.67, 127.30, 127.12, 127.06, 125.97, 125.90, 125.85, 125.72, 125.62, 125.05, 124.21, 122.96, 122.89, 121.49, 121.06, 115.93, 115.02, 114.98, 114.64, 114.20, 111.52, 111.38. MS (ES^+^, 20 V), *m/z*: 596 ([M + H]^+^). Elemental analysis calcd (%) for C_34_H_16_F_6_N_4_: C, 68.69; H, 2.71; F, 19.17; N, 9.42; Found: C, 68.71; H, 2.73; N, 9.47.

#### 2.2.3. 4,4′-(Indolo[3,2-*b*]carbazole-5,11-diyl)bis(3-(trifluoromethyl)benzonitrile) (**3IndCz**)

**3IndCz** was prepared according to the method similar to that used for the preparation of **1IndCz**, except that indolo[3,2-*b*]carbazole (0.5 g, 1.95 mmol), 4-fluoro-3-(trifluoromethyl)benzonitrile (0.81 g, 4.29 mmol) were used. The crude product was purified by column chromatography on silica gel (eluent chloroform/*n*-hexane, 1:2), and the yellow solid was obtained. FW = 594.52 g/mol, yield: 0.90 g, 78%. ^1^H NMR (400 MHz, DMSO, δ, ppm): 8.81 (s, 2H), 8.51 (d, *J* = 8.1 Hz, 2H), 8.24 (d, *J* = 7.7 Hz, 2H), 7.94 (d, *J* = 8.1 Hz, 2H), 7.86 (s, 2H), 7.39 (t, *J* = 7.7 Hz, 2H), 7.24 (t, *J* = 7.7 Hz, 2H), 6.99 (d, *J* = 8.1 Hz, 2H). ^13^C NMR (101 MHz, DMSO, δ, ppm): 143.60, 140.56, 140.54, 139.55, 138.94, 134.89, 133.05, 133.01, 131.33, 131.02, 127.01, 124.15, 123.59, 121.43, 121.22, 120.38, 117.78, 113.78, 110.22, 101.28. MS (ES^+^, 20 V), *m/z*: 595 ([M + H]^+^). Elemental analysis calcd (%) for C_34_H_16_F_6_N_4_: C, 68.69; H, 2.71; F, 19.17; N, 9.42; Found: C, 68.73; H, 2.70; N, 9.45.

### 2.3. Instrumentation

^13^C NMR, ^1^H NMR spectra were obtained using a Unity Inova (Varian, CA, USA) (400 MHz (^1^H) and 101 MHz (^13^C)). Mass (MS) spectra, infrared (IR) spectra were recorded, elemental, thermogravimetric analysis (TGA), differential scanning calorimetry (DSC) measurements, cyclic voltammetry (CV) measurements were performed, absorption, photoluminescence (PL) spectra of dilute solutions and of the films were recorded as described earlier [36]. Melting points of the prepared compounds were estimated by an Electrothermal Melt-Temp 1101D (Dubuque, IA, USA) (apparatus (error value ±1 °C). Theoretical calculations were carried out using Gaussian 16 and Gaussview 6 software. Ionization potential measurements of the solid samples were performed by photoelectron emission method in air. The samples were fabricated by vacuum deposition of the compounds on a pre-cleaned glass/ITO substrate and the aluminum top electrode under vacuum higher than 2 × 10^−6^ mBar. The thicknesses of the layers of **1IndCz**, **2IndCz** and **3IndCz** were 1.65, 2.95 and 4.8 μm, respectively. Hole mobilities (*μ*_h_) were estimated by time of flight technique [37].

The X-ray analysis of the powder samples was performed using D8 Discover X-ray diffractometer (Bruker AXS GmbH, Billerica, MA, USA) with Cu Kα (λ = 1.54 Å) X-ray source. Parallel beam geometry with 60 mm Göbel mirror (i.e., X-ray mirror on a high precision parabolic surface) was used. Primary side also had a Soller slit with an axial divergence of 2.5° and a slit of 1.0 mm. The secondary side had a LYNXEYE (1D mode) detector with an opening angle of 2.16° and slit opening of 6.0 mm. X-ray generator voltage and current were 40.0 kV and 40 mA, respectively. Coupled 2θ/θ scans were performed in the range of 5.0–90.0° with a step size of 0.043° time per step of 19.2 s and auto-repeat function enabled. Processing of the resultant diffractograms was performed with DIFFRAC.EVA software.

OLEDs with indolocarbazoles derivatives as emitters were fabricated by vacuum deposition (at vacuum of ca. 2 × 10^−6^ mBar). The layers of all the devices were deposited at the same time except the light-emitting layers. Pre-cleaned and pre-patterned indium tin oxide (ITO)-coated glass substrates with a sheet resistance of 15 Ω/sq were used for device fabrication. A certificated photodiode PH100-Si-HA-D0 together with the PC-Based Power and Energy Monitor 11S-LINK (STANDA, Vilnius, Lithuania) and Keithley 2400C (Cleveland, OH, USA) source meter were used for collecting current density-voltage and brightness-voltage characteristics. An AvaSpec-2048XL (Avantes, Apeldoorn, The Netherlands) spectrometer was used for recording of electroluminescence (EL) spectra. The characteristics of brightness and current density as a function of voltage together with EL spectra were used for the calculation of device efficiencies.

## 3. Results and Discussion

### 3.1. Synthesis

**Scheme 1:**
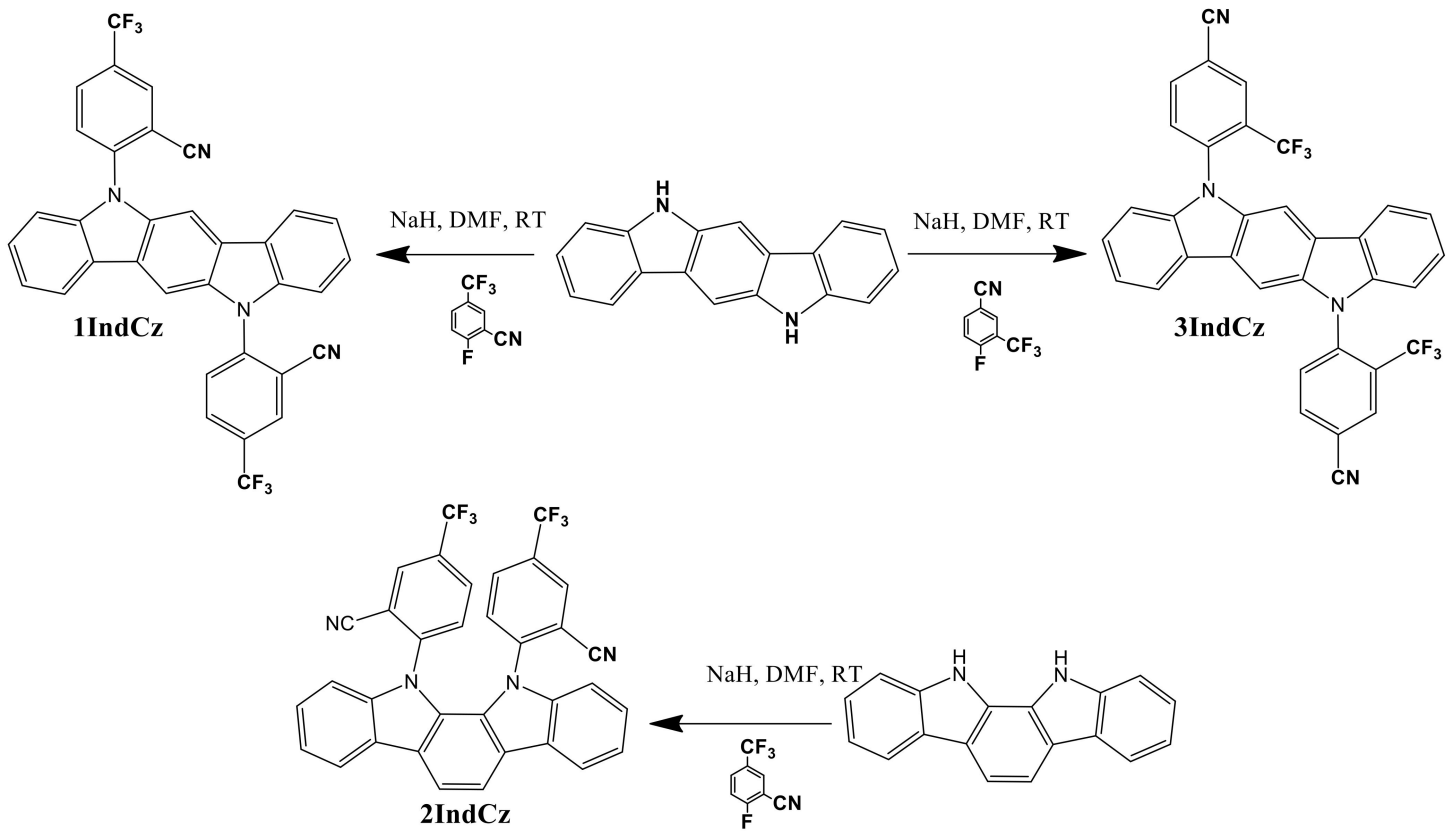
Synthesis of compounds **1IndCz**, **2IndCz** and **3IndCz**.

Indolocarbazole derivatives **1IndCz**, **2IndCz** and **3IndCz** were synthesized by the reactions of indolo[2,3-*a*]carbazole or indolo[3,2-*b*]carbazole with 2-fluoro-5-(trifluoromethyl)benzonitrile or 4-fluoro-3-(trifluoromethyl)benzonitrile in the presence of sodium hydride to offer the target compounds in high yields (Scheme 1). The chemical structures of the target compounds were proved by mass spectrometry, ^1^H NMR, ^13^C NMR spectroscopy and elemental analysis. The compounds were purified by column chromatography.

### 3.2. Theoretical Calculations

**Figure 1:**
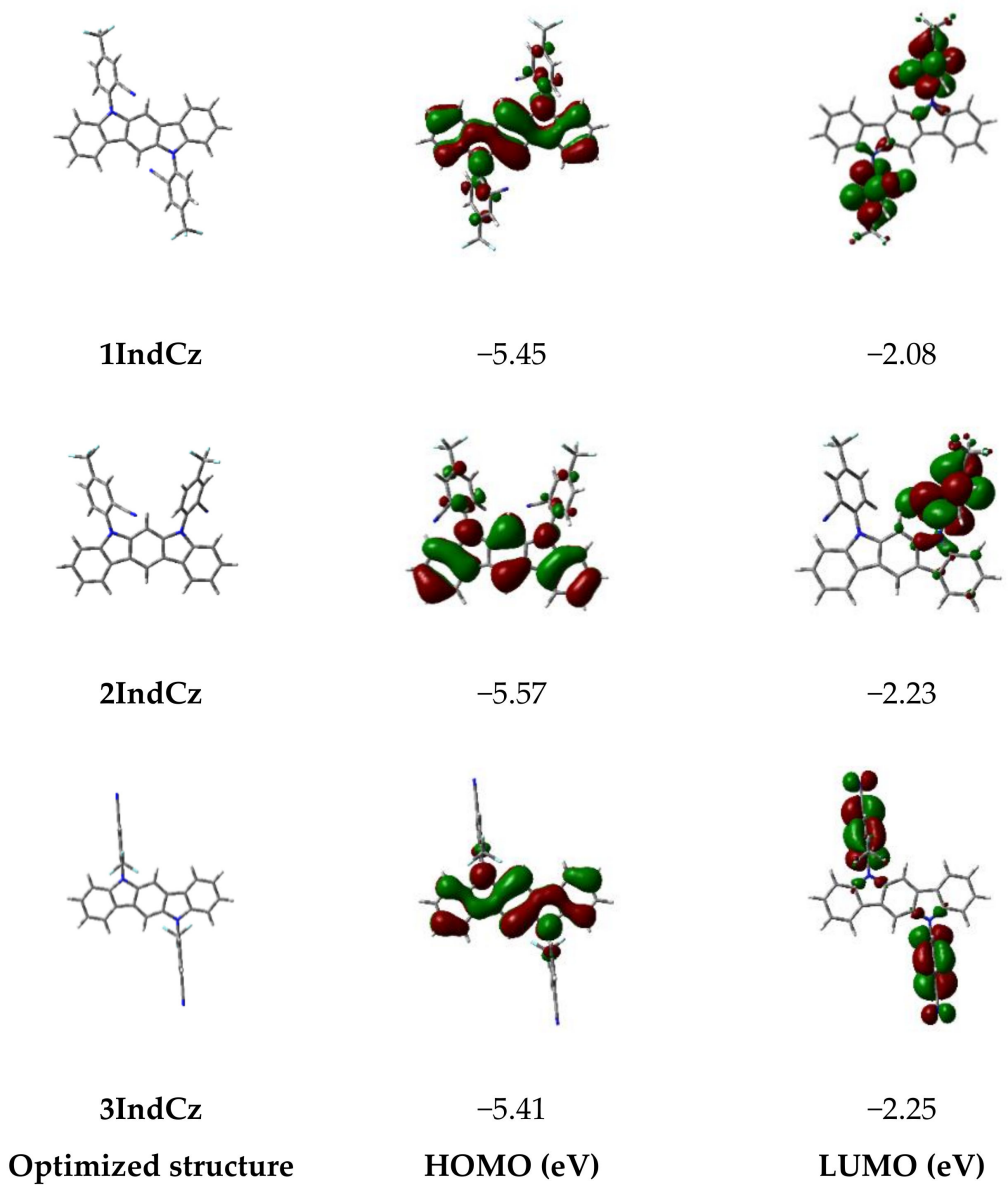
FMO distributions of derivatives **1IndCz**, **2IndCz** and **3IndCz,** calculated by employing the B3LYP/6–31G(d,p) basis set of the Gaussian 16 program. The positive (red) and negative (green) phase distributions in molecular orbital wave function.

To estimate the frontier molecular orbital distributions, singlet-triplet energy splitting (ΔE_ST_) of the compounds, and density functional theory (DFT) calculations were employed, which were carried out by B3LYP method with 6–31G(d,p) basis set. The optimized geometries and the frontier molecular orbital (FMO) distributions of the derivatives are depicted in Figure 1. The HOMO orbitals of derivatives **1IndCz** and **2IndCz** were mainly localized on the indolocarbazole moieties, which means considerable overlap between HOMO and LUMO. This result may determine the high PLQY of **1IndCz** and **2IndCz**. Meanwhile, the HOMO orbitals of **3IndCz** were delocalized over the indolocarbazole moieties and 3-(trifluoromethyl)benzonitrile units. The LUMO orbitals of compounds **1IndCz**, **2IndCz** and **3IndCz** were mainly localized on 3-(trifluoromethyl)benzonitrile moieties.

The time-dependent DFT calculations were carried out based on the optimized ground state geometries. The singlet energy levels of **1IndCz**, **2IndCz** and **3IndCz** were calculated to be 2.78, 2.62 and 2.54 eV, respectively. The triplet energy levels of **1IndCz**, **2IndCz** and **3IndCz** were calculated to be 2.61, 2.61 and 2.53 eV, respectively. Thus, the corresponding ΔE_ST_ values are 0.17, 0.01 and 0.01 eV, respectively.

### 3.3. Thermal Characterization

**Figure 2:**
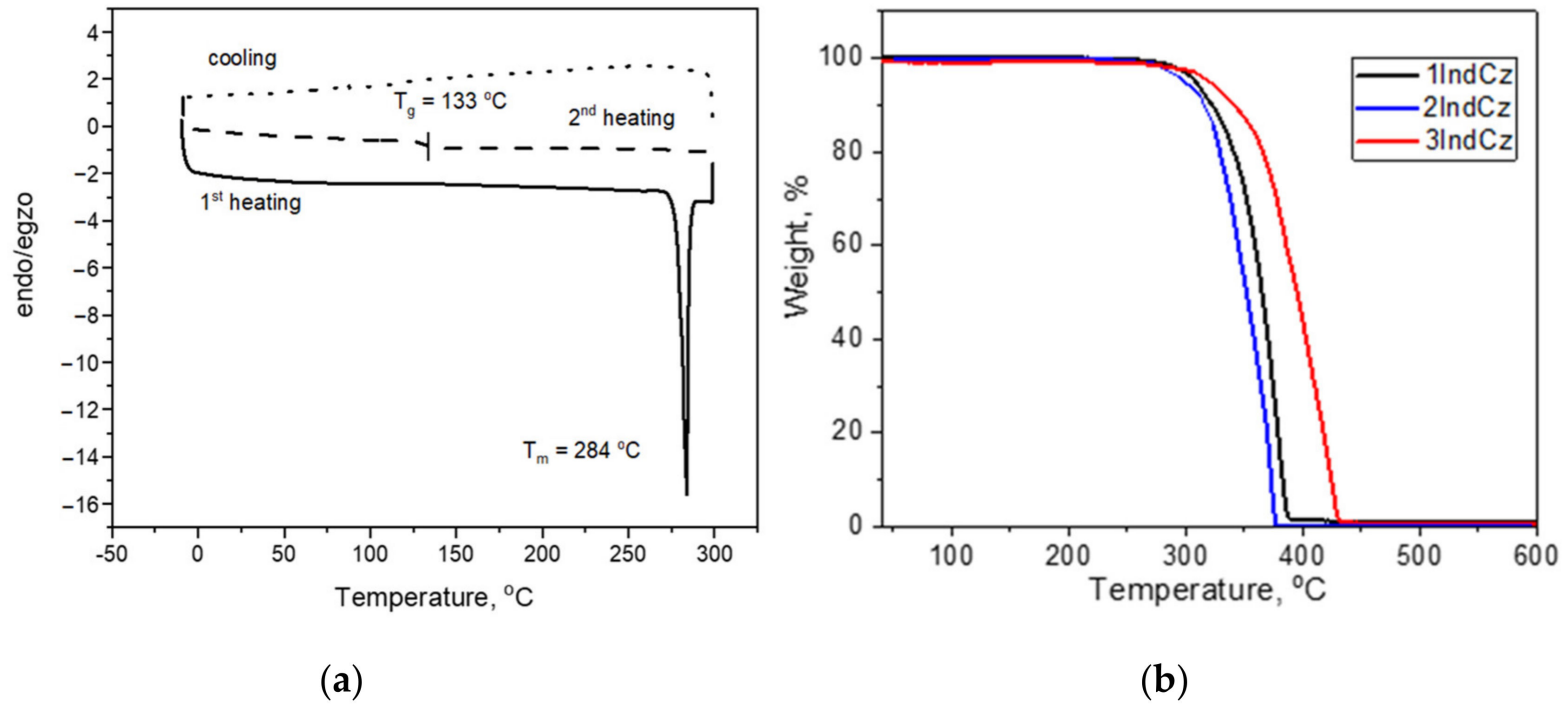
(**a**) DSC thermograms of compound **2IndCz**; (**b**) TGA curves of **1IndCz**, **2IndCz** and **3IndCz**.

The behavior under heating of the compounds was studied by DSC and TGA (Figure 2). The data are displayed in Table 1. The investigated compounds exhibited relatively high thermal stability. The 5% weight loss temperatures (T_d−5%_) ranged from 301 to 344 °C (Figure 2). The complete weight loss in the single stage of the compounds showed that the reason for weight loss was sublimation.

When a crystalline sample of **2IndCz** was heated, the melting temperature was observed by DSC at 284 °C. When the melted sample was cooled down, it was transformed into an amorphous material with a glass transition temperature (T_g_) of 133 °C (Table 1). The DSC measurements confirmed that compounds **1IndCz**, **3IndCz** were obtained as amorphous substances. They showed glass transitions at 70 and 87 °C, respectively. The T_g_ value of **2IndCz** containing indolo[3,2-*a*]carbazole moiety was found to be considerably higher compared to those of **1IndCz** and **3IndCz** based on indolo[2,3-*b*]carbazole. A higher T_g_ of derivatives of indolo[3,2-*a*]carbazole can apparently be attributed to the enhanced rigidity of the molecules due to closely located benzonitrile moieties.

### 3.4. Photophysical Properties

**Figure 3:**
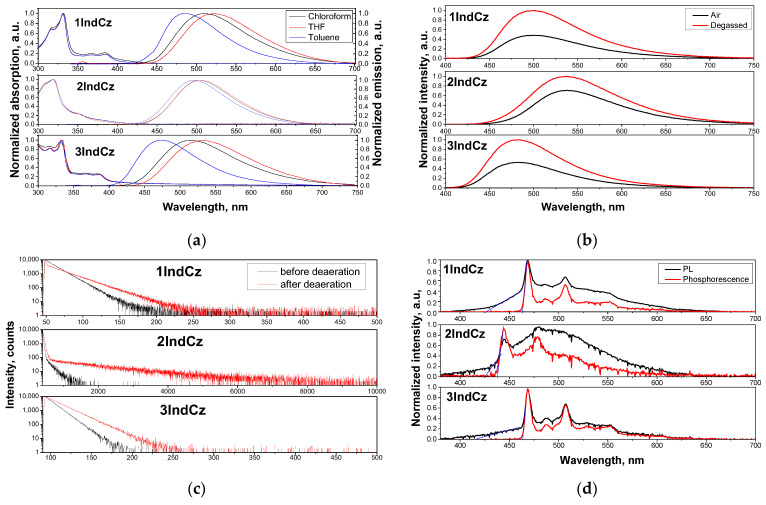
Absorption and PL spectra (**a**) of dilute solutions of **1IndCz**, **2IndCz** and **3IndCz** in the different solvents; PL spectra (**b**) and PL decay curves (**c**) of air equilibrated and deoxygenated solutions of the compounds in toluene; as well as PL and phosphorescence spectra (**d**) recorded at 77 K of THF solutions of **1IndCz**, **2IndCz** and **3IndCz**. The phosphorescence spectra were recorded using a delay of 50 ms after excitation. Excitation wavelengths of 350 nm for PL spectra and 374 nm for PL decay curves were selected. The onsets of PL and phosphorescence are marked by blue lines.

The PL and absorption spectra of the solutions of the studied compounds in solvents of different polarities (toluene (*p* = 0.36 D), chloroform (*p* = 1.04 D) and tetrahydrofuran (THF) (*p* = 1.63 D)) are shown in Figure 3a. The absorption spectra of the solutions in all cases were found to be independent of the solvent polarity. The lowest energy absorption bands for the dilute solutions of **1IndCz**, **2IndCz** and **3IndCz** in all solvents were observed at 385, 352 and 387 nm, respectively.

Unlike the absorption spectra, the PL spectra of the solutions of the compounds showed a clear dependence on the solvent polarity. The wavelengths of maxima of the PL spectra varied from 492 to 501 to 480 nm for the dilute solutions of **1IndCz**, **2IndCz**, **3IndCz,** respectively, in toluene (the solvent with the lowest polarity) to 528, 509, 543 nm in THF (the highest polarity solvent) (Figure 3a and Table 2). This observation shows that there is a significant difference in dipole moment between the ground and excited state of the studied molecules, which is typical for D-A-type molecules with a small overlap of HOMO and LUMO [38].

To examine the input of the triplet emission, the PL spectra and PL decay curves of the solutions in toluene were recorded before and after deoxygenation (Figure 3b). Both the air-equilibrated and deoxygenated solutions of all the studied compounds exhibited similar wavelengths and shapes to the PL spectra. This observation indicates that the emission (fluorescence) originated from the same excited singlet state. The PL intensities of the deoxygenated solutions of **1IndCz**, **2IndCz** and **3IndCz** in toluene were by factors of 2.08, 1.43 and 1.92, respectively, higher than those of the corresponding air-equilibrated solutions (Figure 3b). This observation confirms the presence of triplet emission when triplets are not deactivated by triplets of oxygen. Participation of triplets can also be recognized via the comparison of the PL decay curves of deoxygenated and air-equilibrated toluene solutions of **1IndCz**, **2IndCz** and **3IndCz** (Figure 3c). The intensity of the long-living emissions was significantly enhanced after deoxygenation of the samples, especially in the case of **2IndCz**. As could be predicted, by taking into account the twisted donor–acceptor molecular structures, such an enhancement of long-living emissions can be explained by the TADF effect [39]. In order to support this assumption, temperature-resolved measurements of the emissions of the solutions THF were performed (Figure 3d). At 77 K, THF becomes solid and geometric relaxation of the solvent is no longer possible. The energies of the lowest singlet (S_1_) and triplet (T_1_) levels were estimated from the onset potentials of photoluminescence and phosphorescence spectra, respectively. The S_1_ values of **1IndCz**, **2IndCz** and **3IndCz** were practically the same (2.91, 2.92 and 2.95, respectively). Meanwhile, their T_1_ values were rather different, as affected by the substituents (2.68, 2.83, 2.68 eV, respectively). As a result, the ΔE_ST_ values of **1IndCz**, **2IndCz** and **3IndCz** were found to be 0.23, 0.09 and 0.27 eV, respectively (Figure 3d, Table 2). The ΔE_ST_ values of **1IndCz** and **3IndCz** were found to be higher than those of typical TADF materials [40], while the ΔE_ST_ of **2IndCz** was found to be lower than 0.1 eV which indicated the higher probability of TADF. This observation is in very good agreement with the results of the PL decay measurements of the deoxygenated and air-equilibrated toluene solutions (Figure 3c).

**Figure 4:**
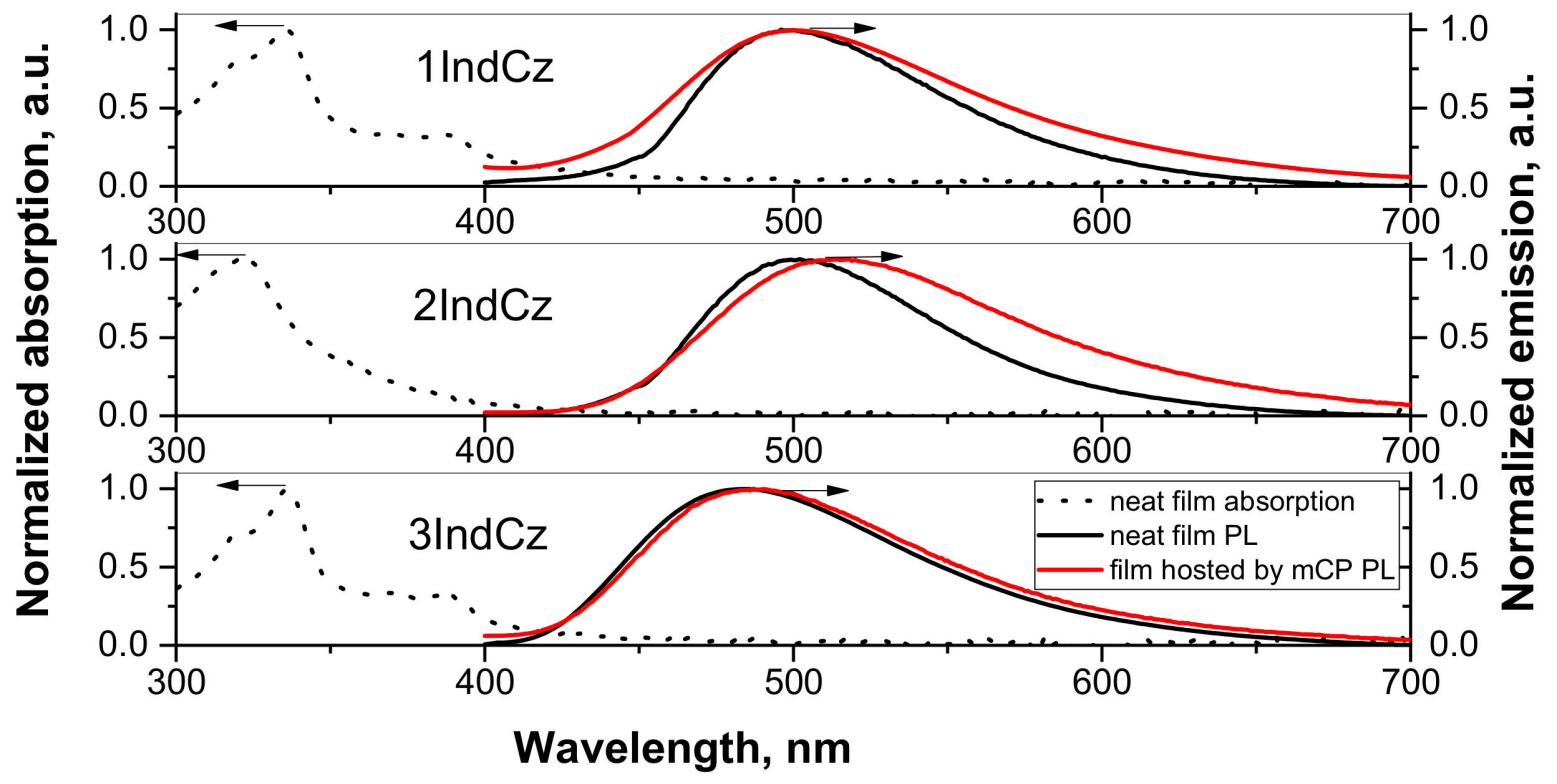
Absorption and PL spectra of pure films and hosted by mCP.

The PL and absorption spectra of the solid films of the studied compounds are shown in Figure 4. The lowest energy bands of the layers of **1IndCz**, **2IndCz** and **3IndCz** were observed at 388, 356 and 388 nm, respectively. The small differences from the spectra of dilute solutions can be explained by the effect of aggregation. The PL spectra of pure solid samples of **1IndCz**, **2IndCz**, **3IndCz** showed emission maxima at 499, 500, 483 nm, respectively (Figure 4 and Table 2). The PL spectra of the films of 20 wt% solid solutions of **1IndCz** and **3IndCz** in mCP showed emission maxima at 501 and 485 nm, respectively, i.e., they were close to those of neat films. However, the PL spectrum of **2IndCz** molecularly dispersed in mCP was a little redshifted. Its maximum was observed at 514 nm (Figure 4 and Table 2). This observation may be explained by either aggregation or solvatochromic-like effects because of the different dielectric constants of the hosts, emitters and solvents which were used in the sample preparation.

Air equilibrated dilute solutions of **1IndCz**, **2IndCz** and **3IndCz** in toluene showed PLQY values of 38, 20 and 35%, respectively. These values are considerably higher than those of the corresponding dilute THF solutions (5, 10 and 6%, respectively). Apparently, under an increase in the polarity of the solvent, non-radiative decay rates increase. After deoxygenation, PLQY values increased up to two times. This observation indicates the presence of emissions originating from the triplet states. The PLQY values of neat films **1IndCz**, **2IndCz** and **3IndCz** were found to be 40, 58 and 31%, respectively. These values are 1.3–3.6 times higher than those of the films of the corresponding compounds doped in mCP. Such a difference between PLQY values of the layers of pure compounds and of their molecular dispersions in mCP indicates the manifestation of AIEE effect resulting from the restriction of intramolecular rotation of acceptor-substituted phenyl groups via single-bonds.

**Figure 5:**
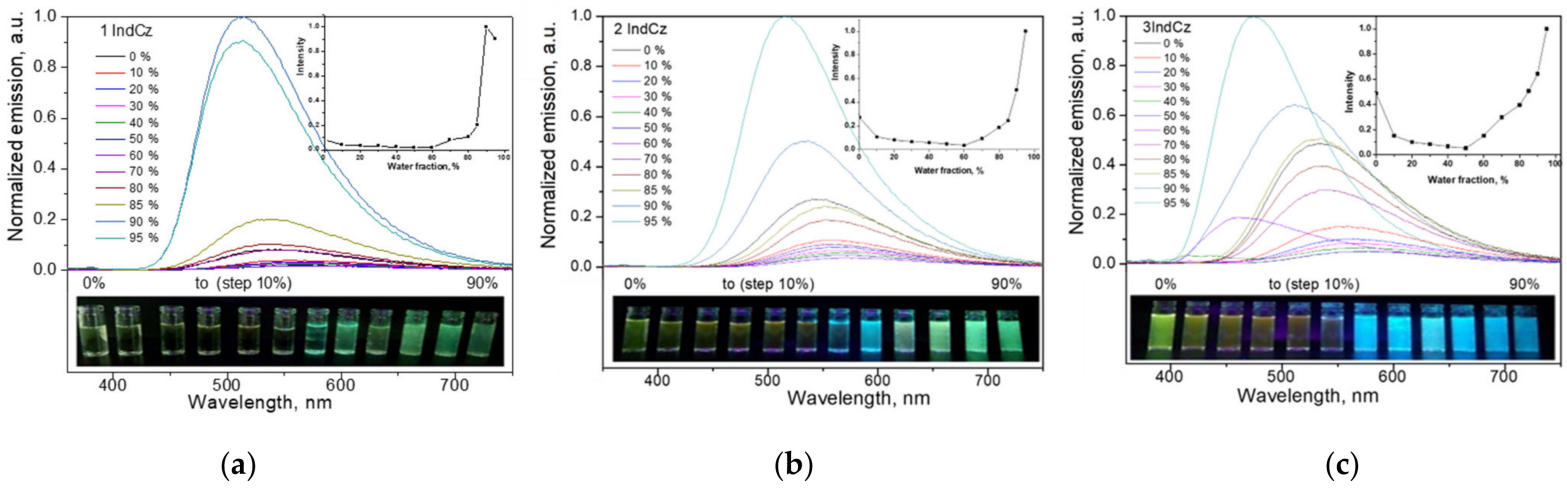
Emission spectra of the dispersions of **1IndCz** (**a**)**, 2IndCz** (**b**) and **3IndCz** (**c**) in THF/H_2_O mixtures with different fractions of water. Inset graphs show dependencies of PL intensity as a function of fractions of water. Inset photos show fluorescence color images of the dispersions in the THF/H_2_O mixtures under UV light.

In order to examine the effect of aggregation on the emissions of **1IndCz**, **2IndCz** and **3IndCz**, the fluorescence spectra of the dispersions of the compounds in THF-water mixtures with differing water fractions were recorded. The images of the dispersions of the compounds in THF-water mixtures with the various water fractions under UV illumination are shown in Figure 5.

Up to a water fraction of 60%, the emission intensity of **2IndCz** and **3IndCz** continuously decreased with the increase in water fraction (Figure 5). However, the PL intensity started to increase with the further increase in water fraction above 60%. Compound **1IndCz** showed a similar pattern, but at the high (80%) water fraction, the intensity of the fluorescence started to decrease (Figure 5a). The reason for this observation is apparently the formation of large aggregates. The emission peaks experienced some bathochromic shifts with the increasing water fraction when the water fraction in the mixture was small (0–40%). This observation can be explained by the increase in the polarity of the THF-water mixtures with the increase in water fraction. However, when the water fraction reached 50%, hypsochromic shifts were observed. The addition of water apparently resulted in the formation of aggregates because of the hydrophobicity of **1IndCz**, **2IndCz** and **3IndCz**. The different colors of the emissions of the different aggregates could be recognized. For example, aggregates of **2IndCz** emit blue or green emissions in TFH/water (70%) or TFH/water (80%) dispersions, respectively (Figure 5b, inset photo). This observation can be attributed to the different molecular assembly in the solid state leading to the formation of different conformers of compounds. If so, it can be presumed that the developed indolocarbazole derivatives would allow their emission properties to be governed via external stimuli.

### 3.5. Mechanochromism of Photoluminescence

**Figure 6:**
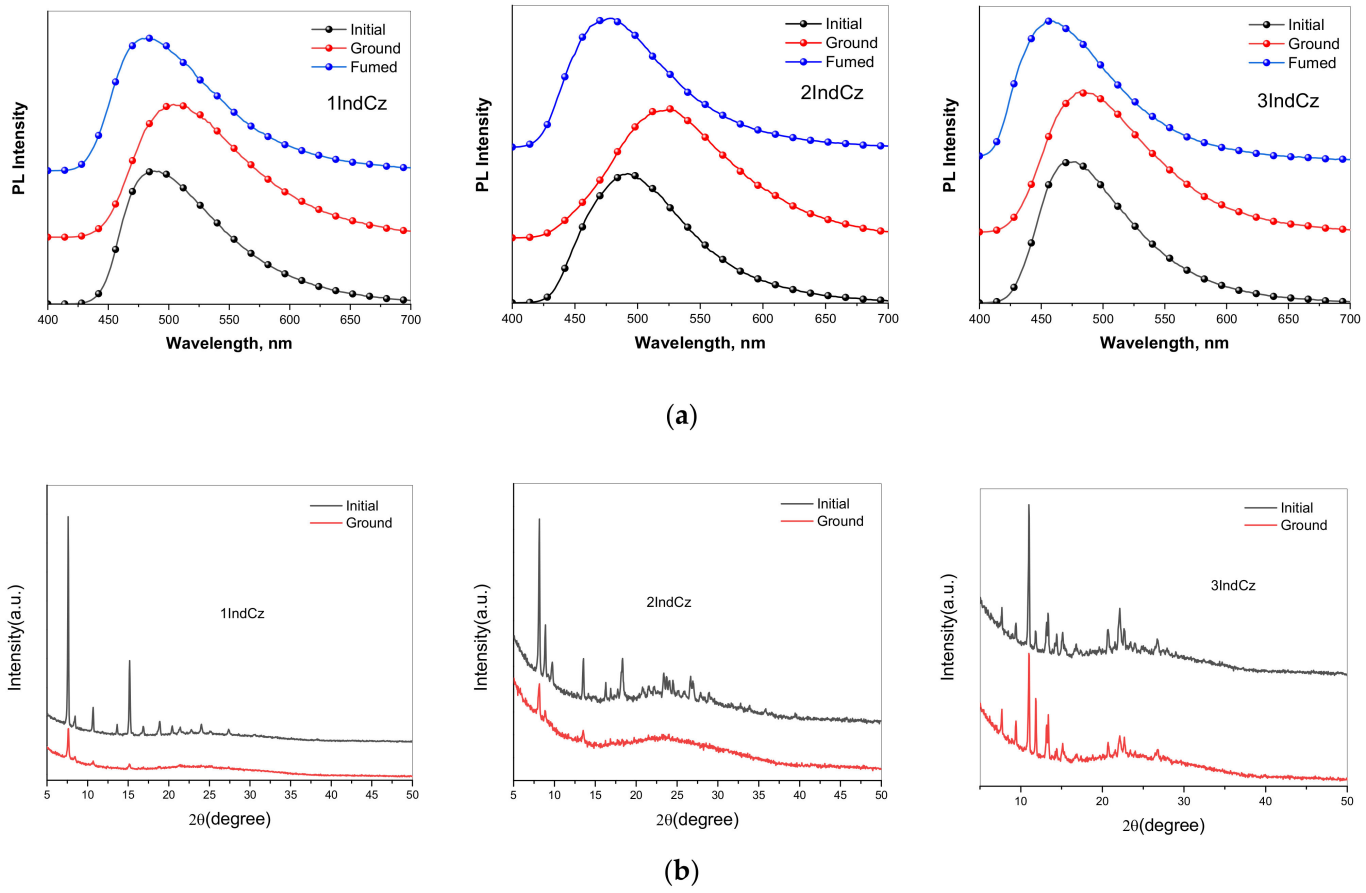
Normalized fluorescence spectra (**a**) and powder XRD patterns (**b**) of compounds in the different solid states.

As was expected, the three synthesized indolocarbazole derivatives exhibited reversible mechanochromic (MC) luminescence through a grinding and solvent fuming treatment (Figure 6a). As-prepared (initial) powders of compounds **1IndCz**, **2IndCz** and **3IndCz** showed sky-blue emissions characterized by PL spectra peaks at 481, 475, 456 nm which redshifted after grinding to 508, 524, 485 nm, respectively. This observation can be explained by the different structures of the initial and ground powders (Figure 6b). The degrees of crystallinity and the crystallite sizes of the initial and ground samples are collected in Table 3. Because of the sterically non-restricted electron-accepting substituents, the color shifts were expected in a wide range. However, the emission spectra of **1IndCz**, **2IndCz** and **3IndCz** were restricted to a narrow range of 27, 49, 29 nm, respectively. It is likely that the incorporation of electron-accepting substituents acted as anchors, resulting in a decrease in the formation of various conformers [33,34].

### 3.6. Electrochemical, Photoelectrical and Charge-Transporting Properties

**Figure 7:**
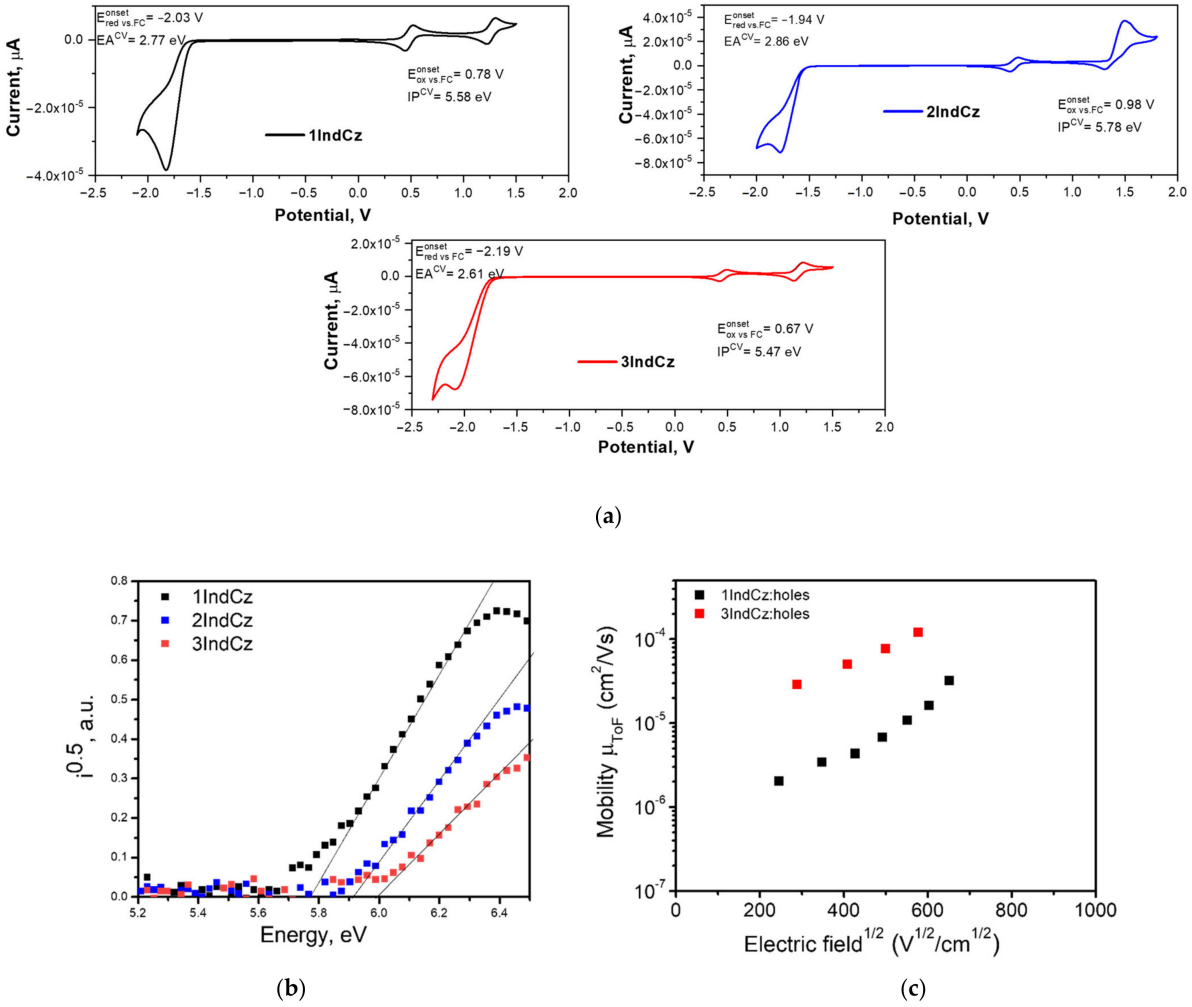
Cyclic voltammograms (**a**) of **1IndCz**, **2IndCz** and **3IndCz** (scan rate of 50 mV/s); electron photoemission spectra (**b**) and electric field dependencies of hole drift mobilities (**c**) of vacuum-deposited layers of **1IndCz**, **2IndCz** and **3IndCz**.

The electrochemical properties of the derivatives of the indolocarbazoles were examined by cyclic voltammetry (CV). The CV curves are shown in Figure 7a. The electrochemical characteristics of **1IndCz**, **2IndCz** and **3IndCz** are collected in Table 3. The oxidation onsets of **1IndCz**, **2IndCz** and **3IndCz** were observed at 1.20, 1.35 and 1.11 V, respectively. Using the oxidation potentials, the ionization potentials (IP^CV^) were calculated by the equation: IP^CV^ = ($E_{\mathrm{onset}}^{\mathrm{ox}}$+ 4.8) (eV). They were found to be 5.58, 5.78, 5.47 for **1IndCz**, **2IndCz**, **3IndCz**, respectively (see Figure 7a). Similarly, using the reduction potential values and the equation EA^CV^ = −$(E_{\mathrm{onset}}^{\mathrm{red}}$+ 4.8) (eV), the electron affinities (EA^CV^) of the derivatives were found to be in the range of 2.61–2.86 eV.

Ionization potentials (IP^EP^) of the vacuum-deposited layers were estimated from electron photoemission spectra (Figure 7b). The values are collected in Table 3. To find the IP^EP^ of **1IndCz**, **2IndCz** and **3IndCz**, the photocurrent versus photon energy (*hv*) were plotted. The extrapolation of the linear part of the plots to the zero photocurrent gave an IP^EP^ of 5.78, 5.91 and 5.99 eV, respectively. Electron affinities (EA^EP^) of the solid samples were calculated using the following equation EA^EP^= IP^EP^ − E_g_, where E_g_ is the optical band-gap energy. The E_g_ values for **1IndCz**, **2IndCz**, **3IndCz** were estimated from the absorption spectra of their pure solid films. They were found to be 2.73, 2.92, 2.80 eV, respectively. EA^EP^ values of **1IndCz**, **2IndCz** and **3IndCz** were calculated to be 3.05, 2.99 and 3.19 eV, respectively.

The charge-transporting properties of the compounds were studied using the time-of-flight (TOF) technique. Transit times (t_tr_) were obtained from current transients recorded at the different electric fields by applying positive voltages to estimate the hole transport capabilities of the vacuum-deposited films of the compounds. Based on the shapes of the TOF current transients, it can be concluded that the films of the compounds were characterized by the low-dispersity transport of holes. The results of the TOF measurements are presented in Figure 7c and Table 3. The TOF measurements showed that only slight modifications resulted in very different charge-transporting properties. Compounds **1IndCz** and **3IndCz** were capable of transporting holes while **2IndCz** transported neither holes nor electrons. This observation may be explained by the different HOMO-HOMO and LUMO-LUMO overlapping of neighboring molecules in the solid layers.

### 3.7. Electroluminescent Properties

**Figure 8:**
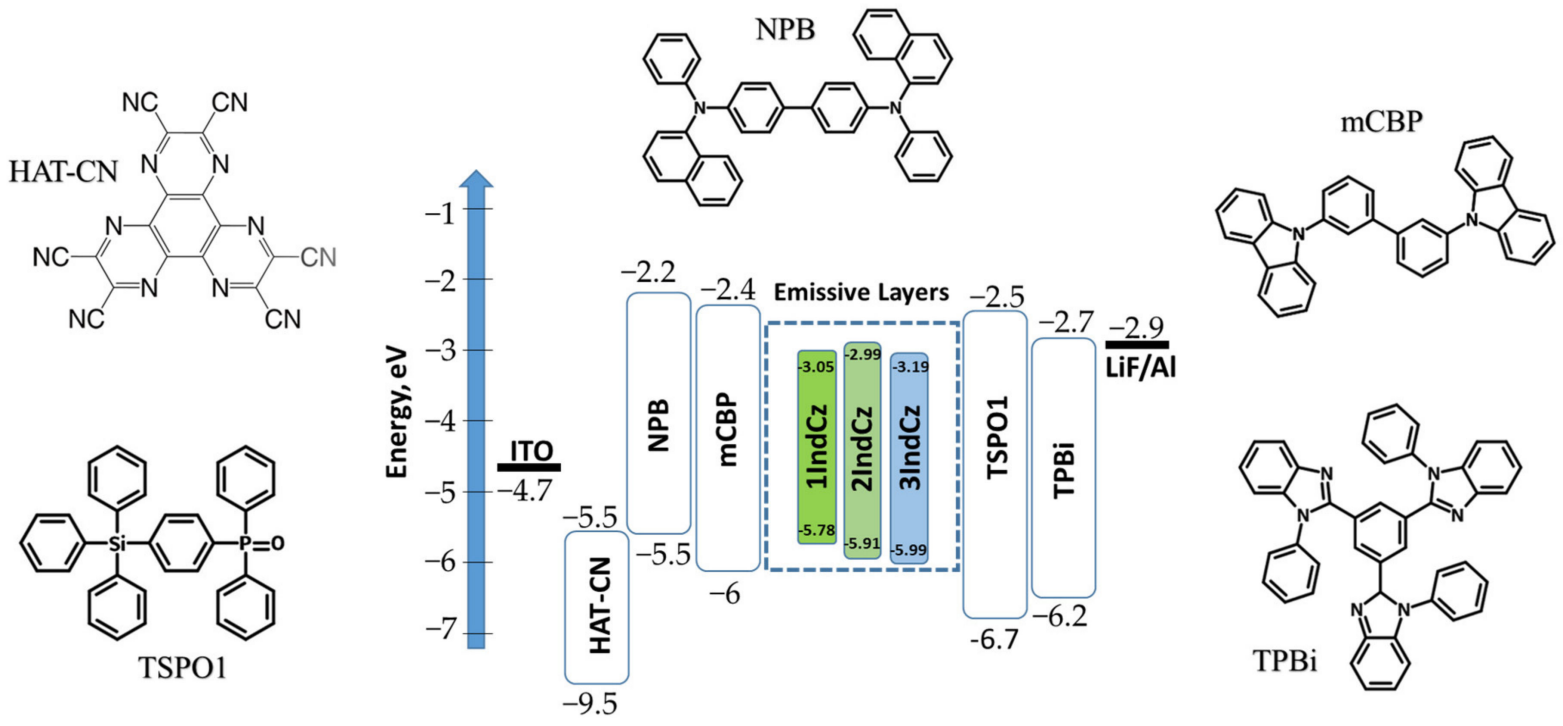
Schematic diagram of the energy levels and molecular structures of the compounds used in devices n1, n2 and n3.

Taking into account the superior properties of **1IndCz**, **2IndCz** and **3IndCz**, electroluminescent devices were fabricated. Since the compounds showed high glass-transition temperatures (T_g_ higher than 70 °C), the vacuum thermal-evaporation method was chosen for device fabrication. The performances of the investigated compounds as emitters were evaluated using device structures of ITO/HAT-CN (8 nm)/NPB (40 nm)/mCBP (8 nm)/IndCz (24 nm)/TSPO1 (8 nm)/TPBi (40 nm)/LiF (0.5 nm)/Al, in which ITO (indium tin oxide) was used as the anode, the layer of 1,4,5,8,9,11-hexaazatriphenylenehex-acarbonitrile (HAT-CN) acted as the hole injection layer, *N*,*N*′-di (1-naphthyl)-*N*,*N*′-diphenyl-(1,1′- biphenyl)-4,4′-diamine (NPB) was used as the hole-transporting material, the layer of 3,3′-di(9H-carbazol-9-yl)-1,1′-biphenyl) (mCBP) was used as the electron blocking layer, **1IndCz, 2IndCz** and **3IndCz** were used for the fabrication emission layers (EML), diphenyl-4-triphenylsilylphenyl-phosphine oxide (mCBP) acted as hole/exciton blocking material, the layer of 2,2′,2″-(1,3,5-benzinetriyl)-tris(1-phenyl-1-H-benzimidazole) (mCBP) acted as the electron-transporting layer, the layer of LiF acted as the electron injection layer and Al was used as the cathode material. The functional layers of the fabricated OLEDs were selected for providing efficient charge injection, charge transport, charge recombination and exciton recombination within the light-emitting layers, taking into account the HOMO/LUMO of all the functional layers. The corresponding HOMO/LUMO are given in the equilibrium energy diagram of the studied host-free devices n1, n2 and n3 shown in Figure 8. The electroluminescence parameters of the fabricated OLEDs are summarized in Table 4.

**Figure 9:**
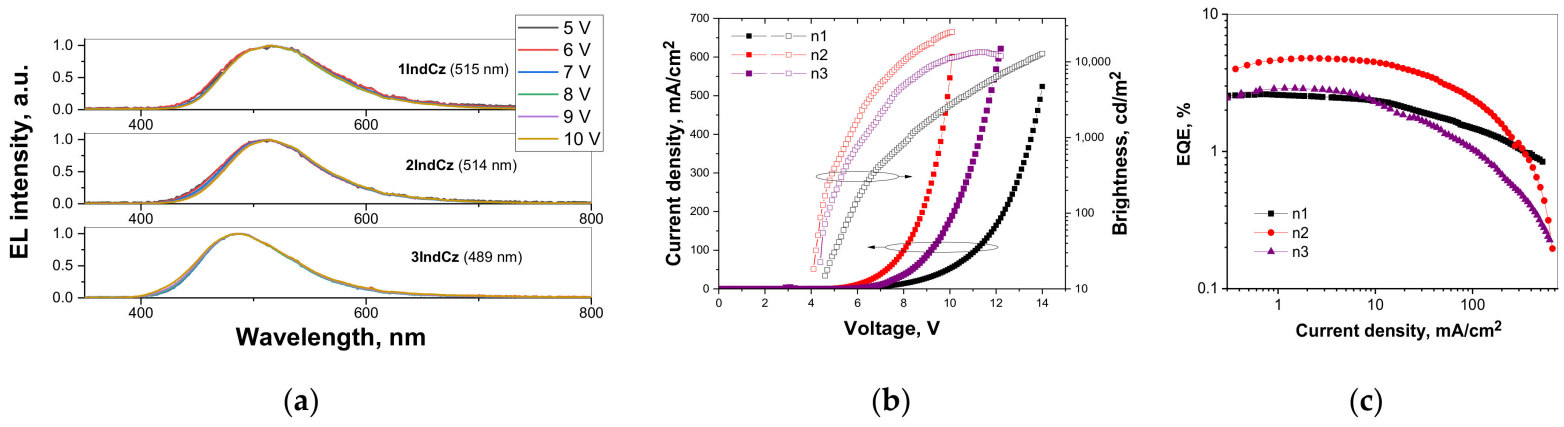
Normalized electroluminescence spectra (**a**) recorded under the different applied voltages (the wavelengths of maxima of EL spectra are shown in brackets); current density/brightness versus applied voltage (**b**), external quantum efficiency versus current density, the oval with an arrow directs to the corresponding (left or right) Y-axis (**c**), for the studied OLEDs.

The electroluminescence (EL) spectra are shown in Figure 9a. Very similar unstructured, broad EL spectra were observed at the different applied voltages for the devices with **1IndCz**, **2IndCz**, **3IndCz** as the emitters. They peaked at 515, 514 and 489 nm, confirming that the recombination of electron–hole pairs occurred within the light-emitting layers. All the fabricated devices showed slight bathochromic shifts of the EL spectra compared with the fluorescence spectra of the vacuum-deposited films. The CIE 1931 chromaticity diagrams of EL are shown in Figure S1. The data are summarized in Table 4. The CIE_x_ and CIE_y_ values observed for the host-free devices ranged from 0.19–0.25 and 0.3–0.47, respectively.

The device efficiencies versus current density curves and current density-voltage-luminance (J-V-L) characteristics are shown in Figure 9b,c and Figure S2. The highest EQE_max_ of 4.8% was observed for device n2 with the **2IndCz** emitter. This value clearly exceeds the theoretical limit of EQE_max_ of 2.9% which can be obtained assuming only conventional fluorescence with a PLQY of 58% observed for the neat film of **2IndCz**. A maximum current efficiency of 12.5 cd/A, maximum power efficiency of 8.8 lm/W and maximum brightness of 24,800 cd/m^2^ were obtained for device n2 based on **2IndCz** (Figure 9b,c and Figure S2, Table 4). The efficient injection of charge carriers from the electrodes and the following transport to the emitting layer was evidenced by relatively low turn-on voltages in all the fabricated OLEDs. The turn-on voltages of the devices were observed in the range from 4.1 to 4.6 V. Device n2 based on **2IndCz** demonstrated the lowest turn-on voltage of 4.1 V. When these compounds were used as hosts for the preparation of guest:host-type light-emitting layers of OLEDs based on the TADF emitter 4,6-di(9,9-dimethylacridan-10-yl)isophthalonitrile [41], the highest EQE of 11% was achieved (see Section: Hosting properties, Supplementary Materials).

## 4. Conclusions

A series of derivatives of indolocarbazoles and trifluoromethyl-substituted benzonitriles were synthesized and characterized. The photophysical, electrochemical, charge-transporting properties of the compounds were studied. Due to aggregation-induced emission enhancement, photoluminescence quantum yields of the neat films of the developed indolocarbazoles reached 58%. Owing to thermally activated delayed fluorescence, the electrically pumped triplets were harvested for electroluminescence allowing a higher external quantum efficiency than the theoretical limit of the emitters, exhibiting prompt fluorescence. Based on the findings of the study on mechanochromic luminescence, it was shown that there is no big effect of conformer formation leading to stable electroluminescence under the different voltages. The ionization potentials of the layers of the compounds established by electron photoemission spectrometry were found to be in the range of 5.78–5.99 eV. The compounds were tested in organic light-emitting diodes as emitters and hosts. The best host-free device, based on 6,6′-(Indolo[2,3-*a*]carbazole-11,12-diyl)bis(3-(trifluoromethyl)benzonitrile) as emitter, showed a turn-on voltage of 4.1 V, maximum brightness of 24,807 cd/m^2^, maximum current efficiency of 12.5 cd/A and external quantum efficiency of ca. 4.8%.

## Figures and Tables

**Table 1:** Thermal characteristics of derivatives **1IndCz**, **2IndCz** and **3IndCz**.

Compound	T_m_, °C ^1^	T_g_, °C ^2^	T_d−5%,_ °C ^3^
**1IndCz**	-	70	313
**2IndCz**	284	133	301
**3IndCz**	-	87	344

^1^ T_m_—melting temperature (scan rate of 10 °C/min, nitrogen atmosphere). ^2^ T_g_—glass-transition temperature, second heating scan. ^3^ T_d_—the temperature of the onset of weight loss (20 °C/min, nitrogen atmosphere).

**Table 2:** Photophysical data of compounds **1IndCz**, **2IndCz** and **3IndCz**.

Compound	Absorption, nm	PL, nm	PLQY, %	S_1_, eV	T_1_, eV	ΔE_ST_, eV
	Toluene/Toluene ^1^/THF/THF ^1^/Film/Hosted Film ^2^			THF		
**1IndCz**	385/-/385/-/388/-	492/-/528/-/499/501	38/68/5/6/40/11	2.91 (2.78)	2.68 (2.61)	0.23 (0.17)
**2IndCz**	352/-/352/-/356/-	501/-/509/-/500/514	20/39/10/15/58/21	2.92 (2.62)	2.83 (2.61)	0.09 (0.01)
**3IndCz**	387/-/387/-/388/-	480/-/543/-/483/485	35/49/6/8/31/10	2.95 (2.54)	2.68 (2.53)	0.27 (0.01)

^1^ Deoxygenated solution. ^2^ mCP host was used. The theoretical values of E_T1_, ΔE_ST_, E_S1_ are depicted in parentheses. Slash punctuation separates different states of media.

**Table 3:** Energy levels, charge mobilities and crystallinity data for **1IndCz**, **2IndCz** and **3IndCz**.

Compound	IP^CV^/IP^EP^, eV	EA^CV^/EA^EP^, eV	μ_h_, cm^2^/Vs	μ_e_, cm^2^/Vs	Degree of Crystallinity, %		Crystallite Size, nm ^1^	
			at 6 × 10^5^ V/cm		Initial	Ground	Initial	Ground
Sample	Solutions/Neat Films		Neat Films		Powders			
**1IndCz**	5.58/5.78	2.77/3.05	1.6 *×* 10^−5^	-	58.5	23.7	72.8	53.8
**2IndCz**	5.78/5.91	2.86/2.99	-	-	44.2	22.7	62.3	37.4
**3IndCz**	5.47/5.99	2.61/3.19	1.2 *×* 10^−4^	-	46.4	41.5	63.1	61.8

^1^ The crystallite size for the reflex with highest intensity.

**Table 4:** Electroluminescence parameters of the fabricated OLEDs.

Device	Light Emitting Layer	V_on_, V ^1^	Max Brightness, cd/m^2^	CE_max_, cd/A ^2^	EQE_max_, % ^3^	λ, nm ^4^	CIE ^5^
Doping-free OLEDs: ITO/HAT-CN/NPB/mCBP/IndCz/TSPO1/TPBi/LiF:Al							
n1	**1IndCz**	4.6	12,861	7.8	2.6	515	(0.25, 0.47)
n2	**2IndCz**	4.1	24,807	12.5	4.8	514	(0.24, 0.47)
n3	**3IndCz**	4.4	13,395	6.8	2.9	489	(0.19, 0.3)

^1^ Turn-on voltage at luminance of 10 cd·m^−2^, ^2^ Maximum current efficiency, ^3^ Maximum external quantum efficiency, ^4^ Wavelength of the peak of EL spectrum at 6 V, ^5^ Commission Internationale de I’Eclairage (CIE) 1931 color coordinates of EL spectra recorded at 9 V.

## Data Availability

Research data may be obtained from the authors on request.

## Supplementary Materials

The following supporting information can be downloaded at: https://www.mdpi.com/article/10.3390/molecules28165999/s1, Figure S1: CIE 1931 color coordinates of fabricated devices at 9V; Figure S2: Current, power and external quantum efficiencies versus current density of OLEDs. Figure S3: Energy diagram of IH devices and molecular structure of the selected TADF emitter DAcIPN. Figure S4: EQE, current density and brightness, electroluminescence spectra at 8V of IH devices. Figure S5: ^1^H, ^13^C NMR and MS spectra of **1IndCz**. Figure S6: ^1^H, ^13^C NMR and MS spectra of **2IndCz**. Figure S7: ^1^H, ^13^C NMR and MS spectra of **3IndCz**. Table S1: Electroluminescent parameters of IH devices; descriptions of hosting properties of **1IndCz**, **2IndCz** and **3IndCz**.

## Sample Availability

Samples of the compounds **1IndCz**, **2IndCz** and **3IndCz** are available from the authors.
