# Supplementary material for: Indolocarbazoles with Sterically Unrestricted Electron-Accepting Anchors Showcasing Aggregation-Induced Thermally Activated Delayed Mechanoluminescence for Host-Free Organic Light-Emitting Diodes
Source: Molecules. 2023 Aug 11;28(16):5999. doi: 10.3390/molecules28165999 (PMC10457976; doi:10.3390/molecules28165999)
Supplement: Supplementary file 1 [file molecules-28-05999-s001.zip › molecules-2527215-supplementary.pdf]

Supporting Information

Indolocarbazoles with sterically unrestricted electron-accepting anchors showcasing aggregation-induced thermally activated delayed mechanoluminescence for host-free organic light-emitting diodes

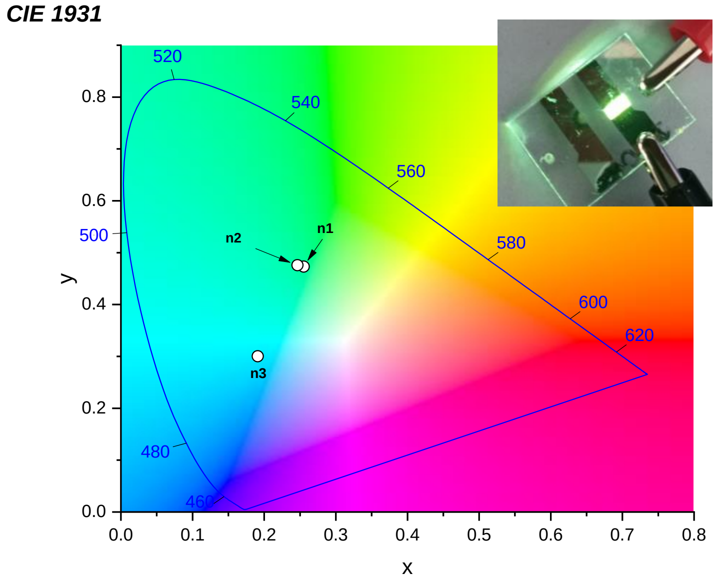

Figure S1. CIE 1931 colour coordinates of fabricated devices at 9V. Inset shows the photo of device n1.

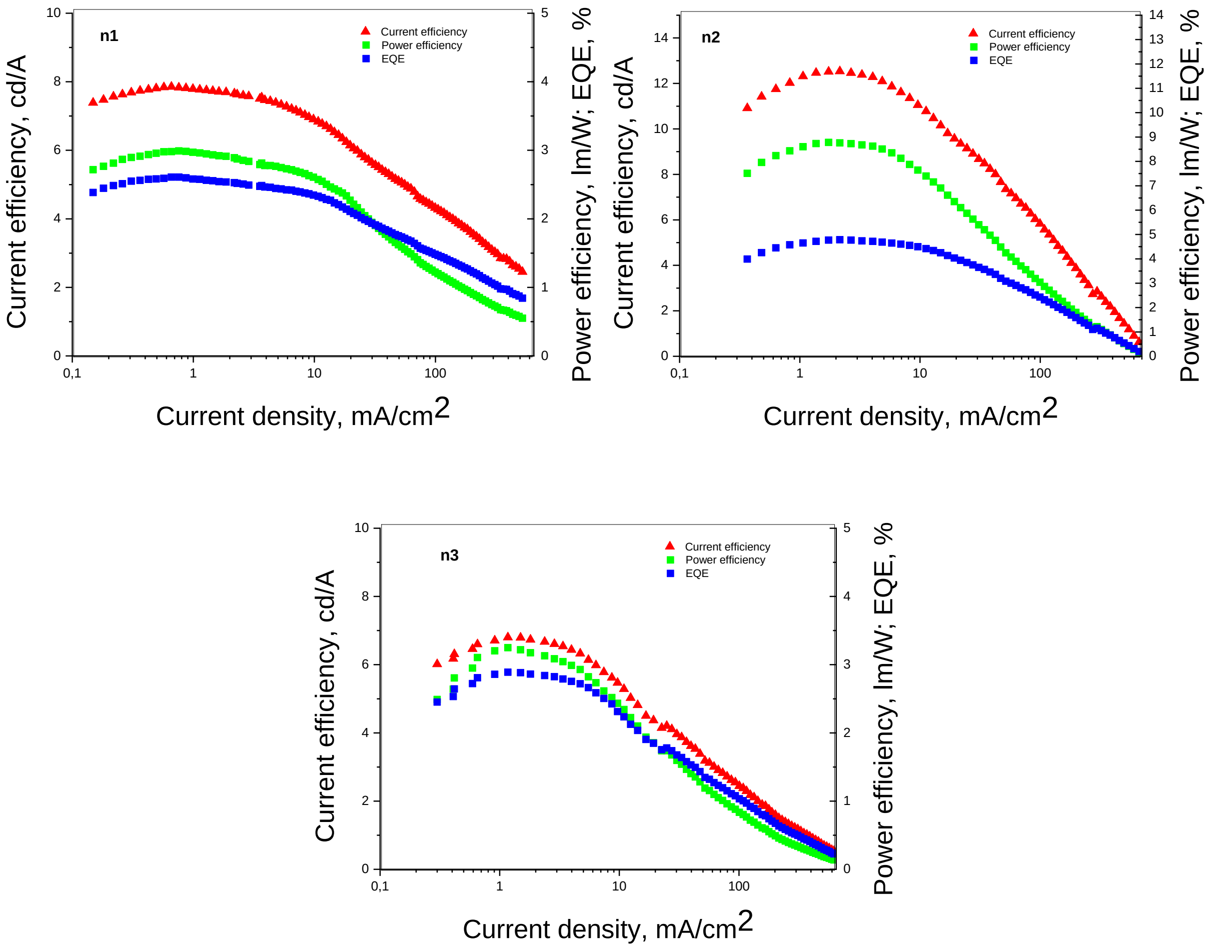

Figure S2. Current, power and external quantum efficiencies versus current density of OLEDs.

Hosting properties

In order to examine hosting properties of **1IndCz**, **2IndCz** and **3IndCz**, OLEDs were designed using the structure of ITO/HAT-CN (8nm)/TAPC (48nm)/mCP (12nm)/**Host:Guest** (24nm)/TSPO1 (12nm)/TPBi (24nm)/LiF:Al (Figure S4a). Host:Guest-type light-emitting layer (EML) of those devices IH1, IH2 and IH3 contained the TADF emitter 4,6-di(9,9-dimethylacridan-10-yl)isophthalonitrile (DAcIPN) (Figure S3b) [S1] and **1IndCz**, **2IndCz**, **3IndCz** as the host. Electroluminescence intensity maxima of IH devices ranged from 545 to 548 nm. These ranges of wavelengths of electroluminescence maxima are close to the wavelengths PL maxima of solid samples of the corresponding emitter DAcIPN [S1]. Comparison of EQE, current efficiency, brightness with current density and electroluminescence spectra at 8V of devices are shown in Figure S4. The main output specifications of devices are collected in Table S1.

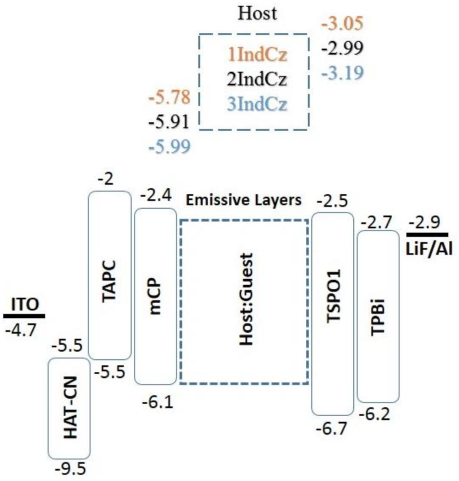

a)

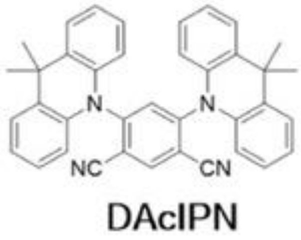

b)

Figure S3. a) Energy diagram of IH devices and b) molecula structure of the selected TADF emitter DAcIPN.

IH3 device had the highest brightness of 9886 cd/m^2 at 9V. The maximum current efficiency of 38.58 d/A, power efficiency of 12.24 lm/W and EQE of 11.09% were recorded for IH3 device.

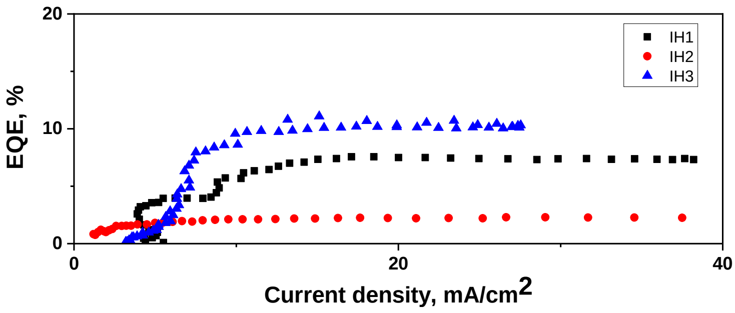

a)

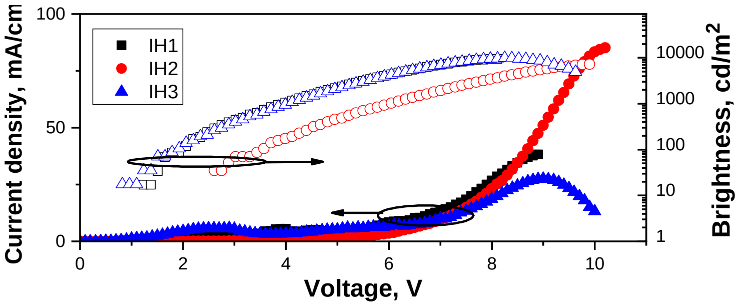

b)

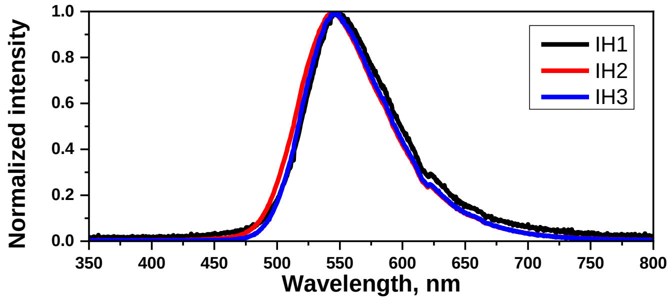

c)

Figure S4. a) EQE, b) Current density and brightness, c) Electroluminiscence spectra at 8V of IH devices.

Table S1. Electroluminescent parameters of IH devices.

Devices	V_{on} [V] at set 20 [cd/m^2]	Current efficiency [cd/A]	Power efficiency [lm/W]	External quantum efficiency [%]	EL maximum [nm]	CIE 1931 UCS coordinates at 8V (x, y)
		at max				
**IH1**	3.96	25.57	10.71	7.56	548	(0.39, 0.57)
**IH2**	3.93	8.51	2.62	2.44	545	(0.37, 0.59)
**IH3**	4.26	38.58	12.24	11.09	548	(0.38, 0.59)

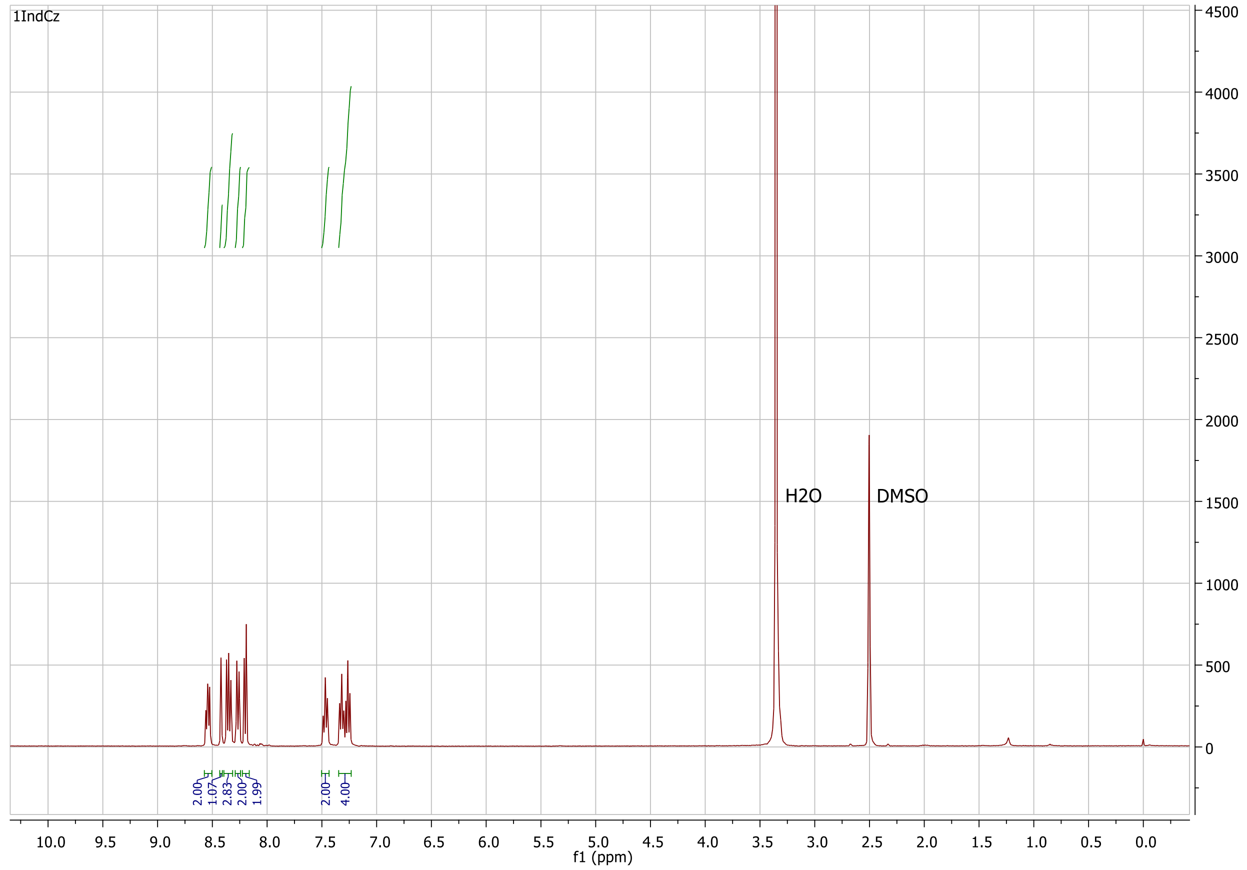

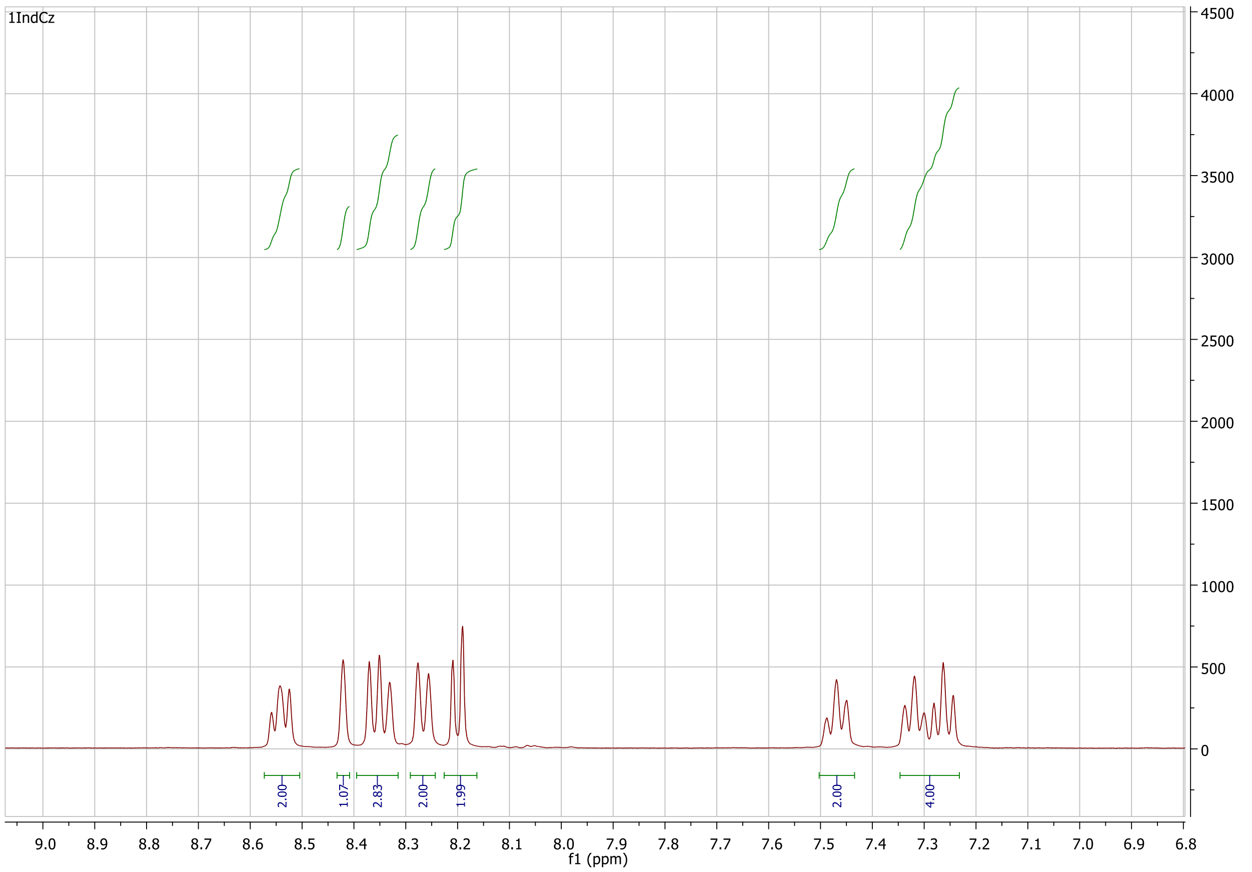
1IndCz
2.00
1.07
2.83
2.00
1.99
2.00
4.00
f1 (ppm)

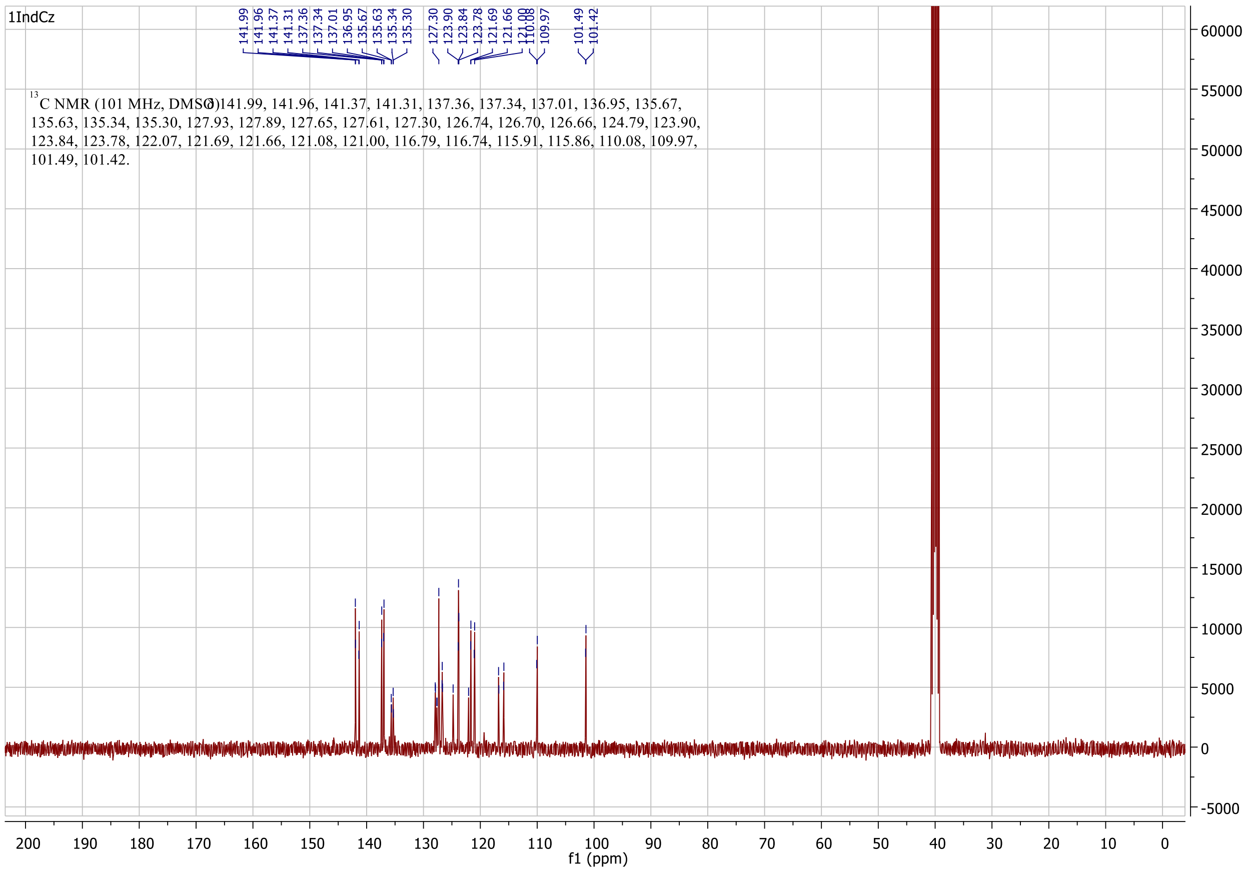
1IndCz
141.99
141.96
141.37
141.31
137.36
137.34
137.01
136.95
135.67
135.63
135.34
135.30
127.30
123.90
123.84
123.78
121.69
121.66
121.08
121.00
110.08
109.97
101.49
101.42
13C NMR (101 MHz, DMSO) δ 141.99, 141.96, 141.37, 141.31, 137.36, 137.34, 137.01, 136.95, 135.67, 135.63, 135.34, 135.30, 127.93, 127.89, 127.65, 127.61, 127.30, 126.74, 126.70, 126.66, 124.79, 123.90, 123.84, 123.78, 122.07, 121.69, 121.66, 121.08, 121.00, 116.79, 116.74, 115.91, 115.86, 110.08, 109.97, 101.49, 101.42.
f1 (ppm)

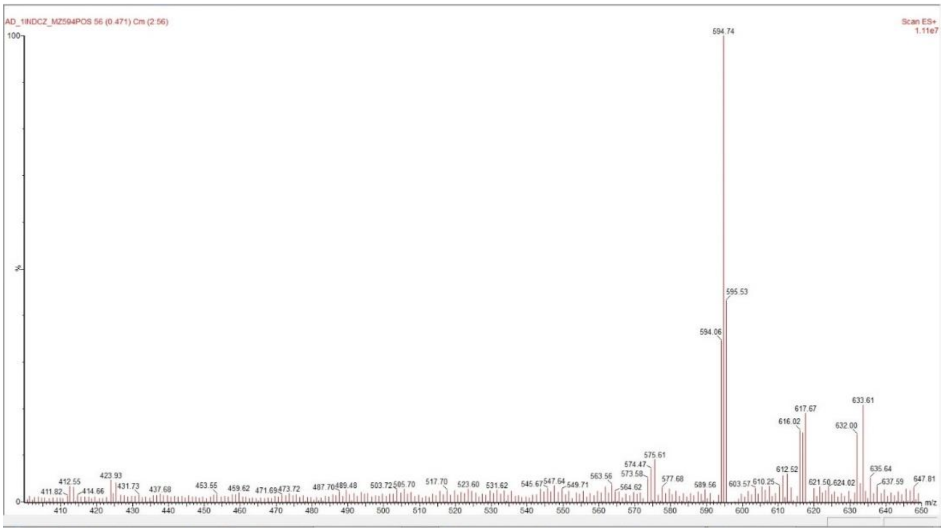

Figure S5. ^{1}H, ^{13}C NMR and MS spectra of 1IndCz.

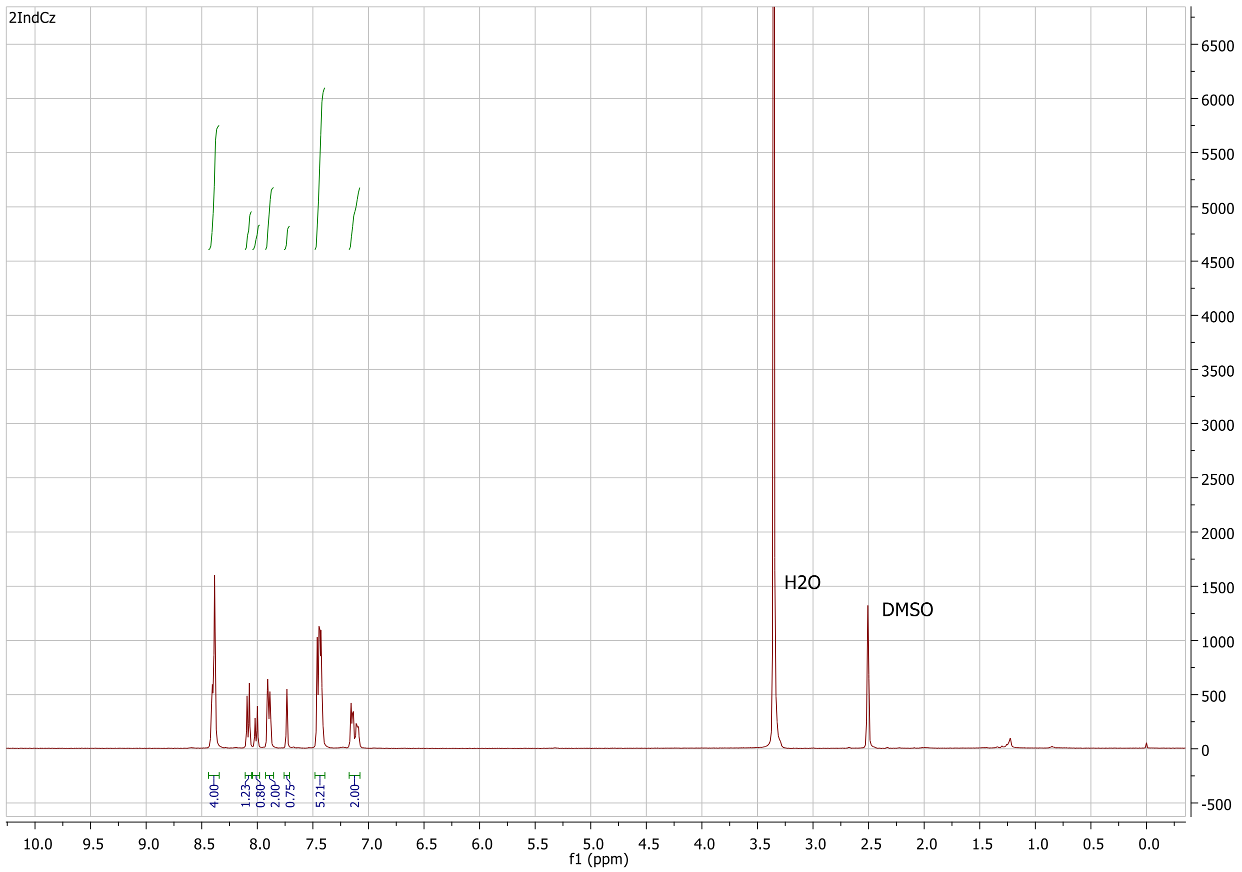
2IndCz
H2O
DMSO
4.00
1.23
0.80
2.00
0.75
5.21
2.00
f1 (ppm)

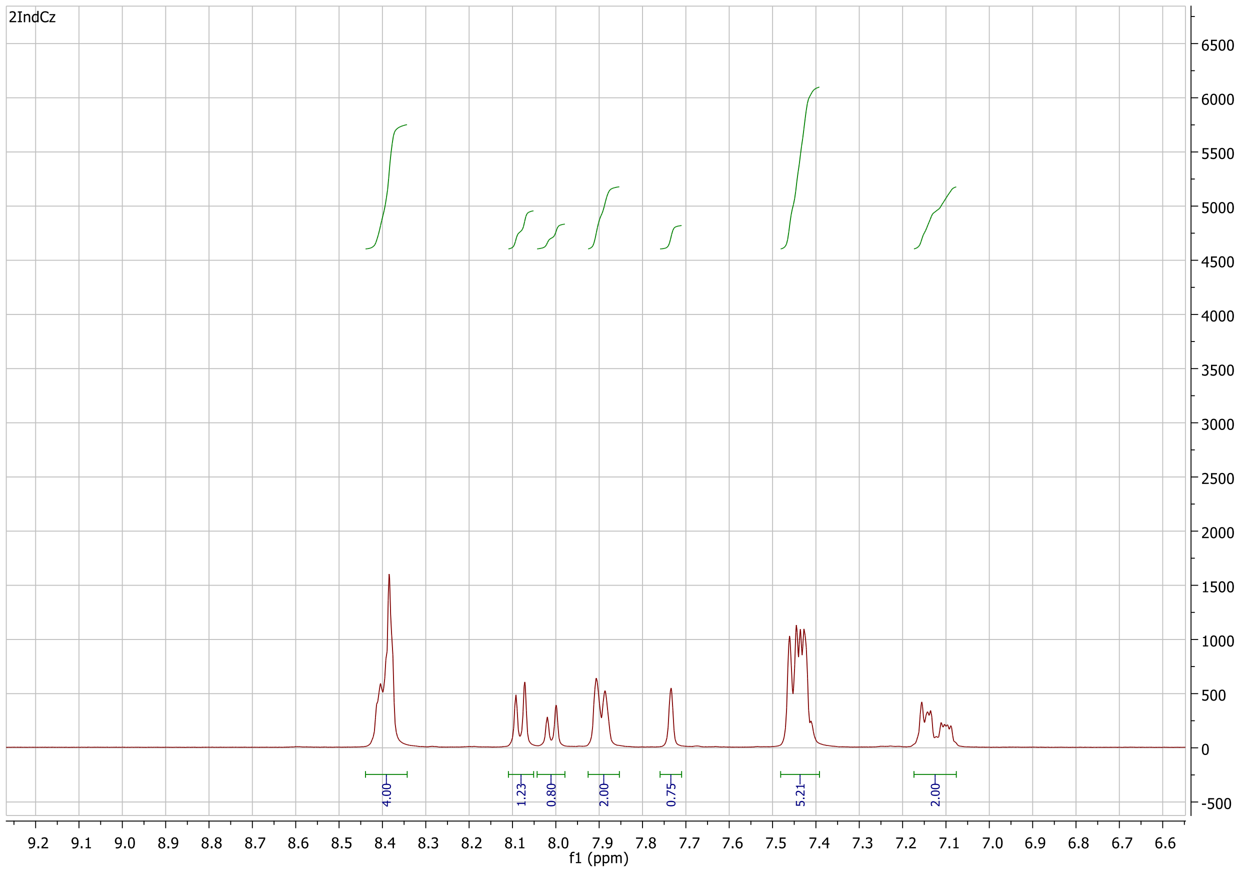
2IndCz
4.00
1.23
0.80
2.00
0.75
5.21
2.00
f1 (ppm)

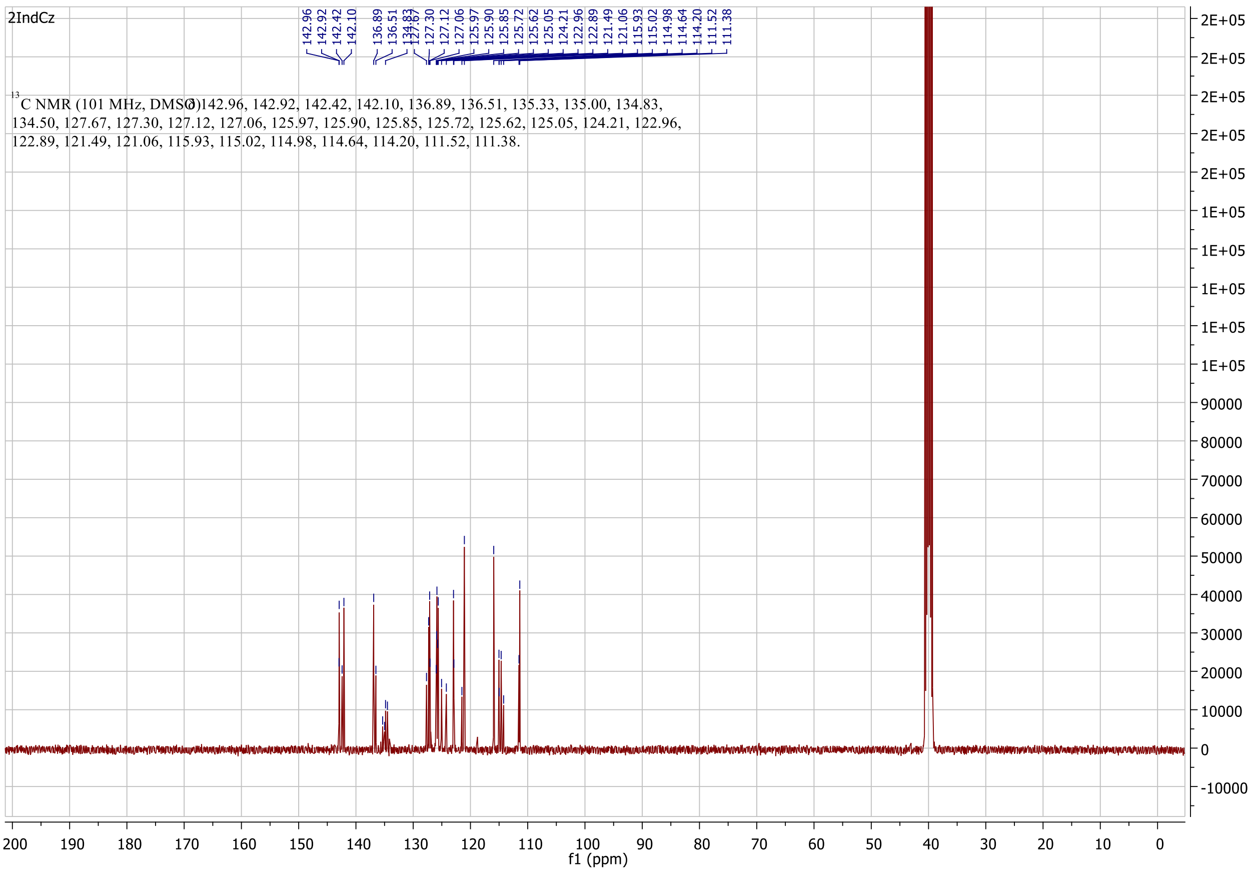

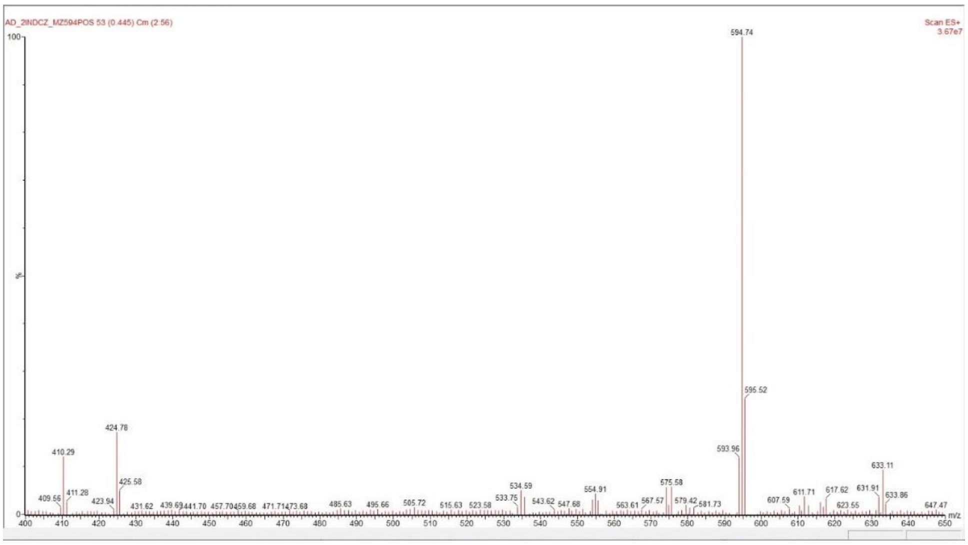

Figure S6. ^{1}H, ^{13}C NMR and MS spectra of 2IndCz.

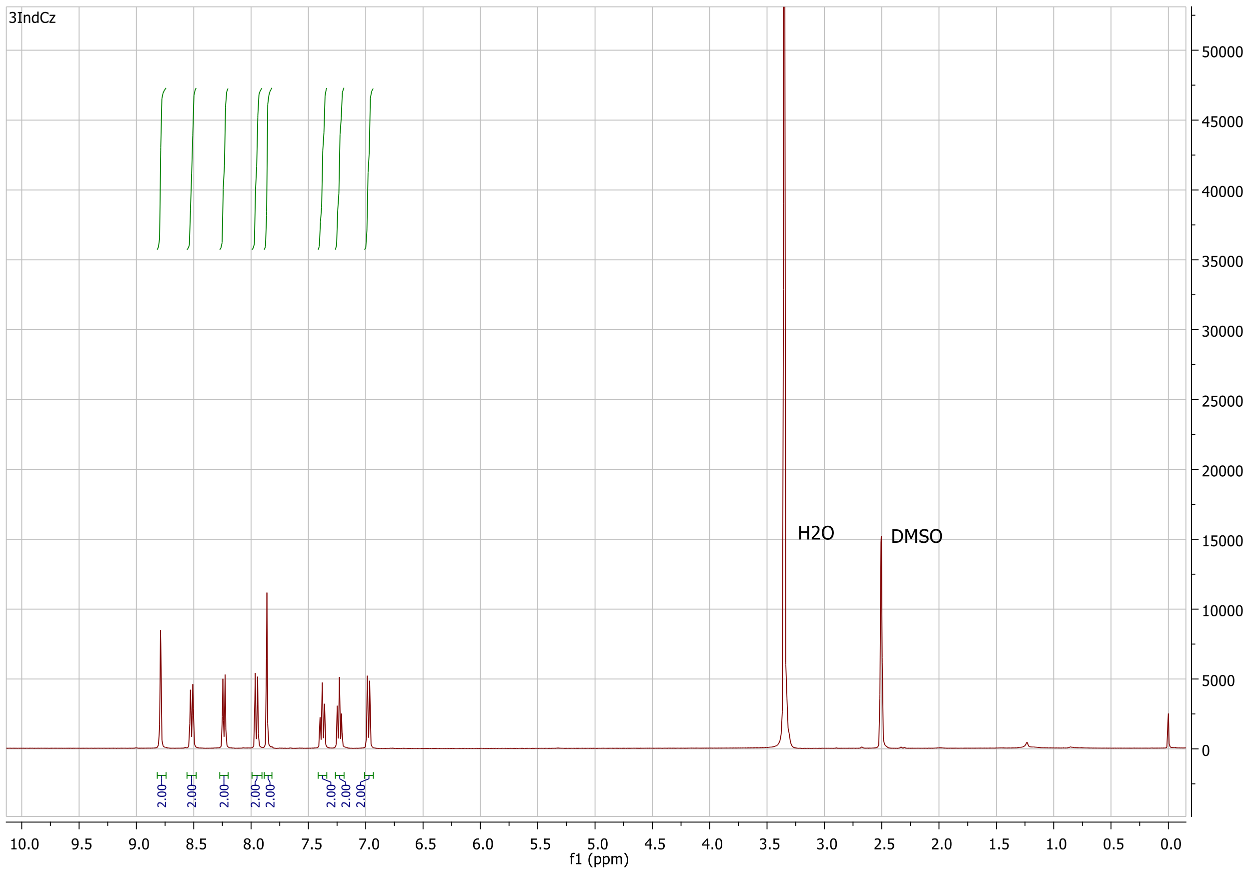
3IndCz
H2O
DMSO
2.00
2.00
2.00
2.00
2.00
2.00
2.00
2.00
f1 (ppm)

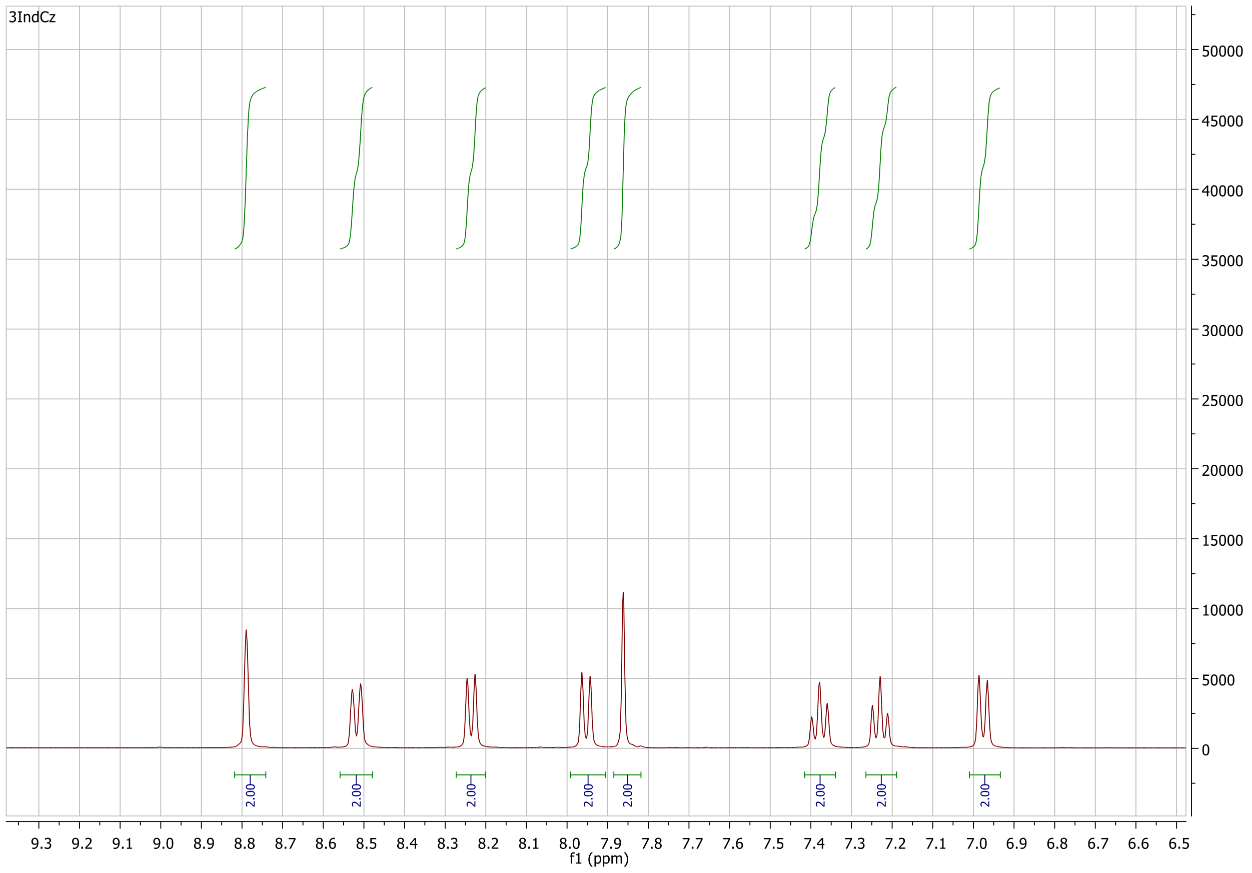
3IndCz
2.00
2.00
2.00
2.00
2.00
2.00
2.00
2.00
f1 (ppm)

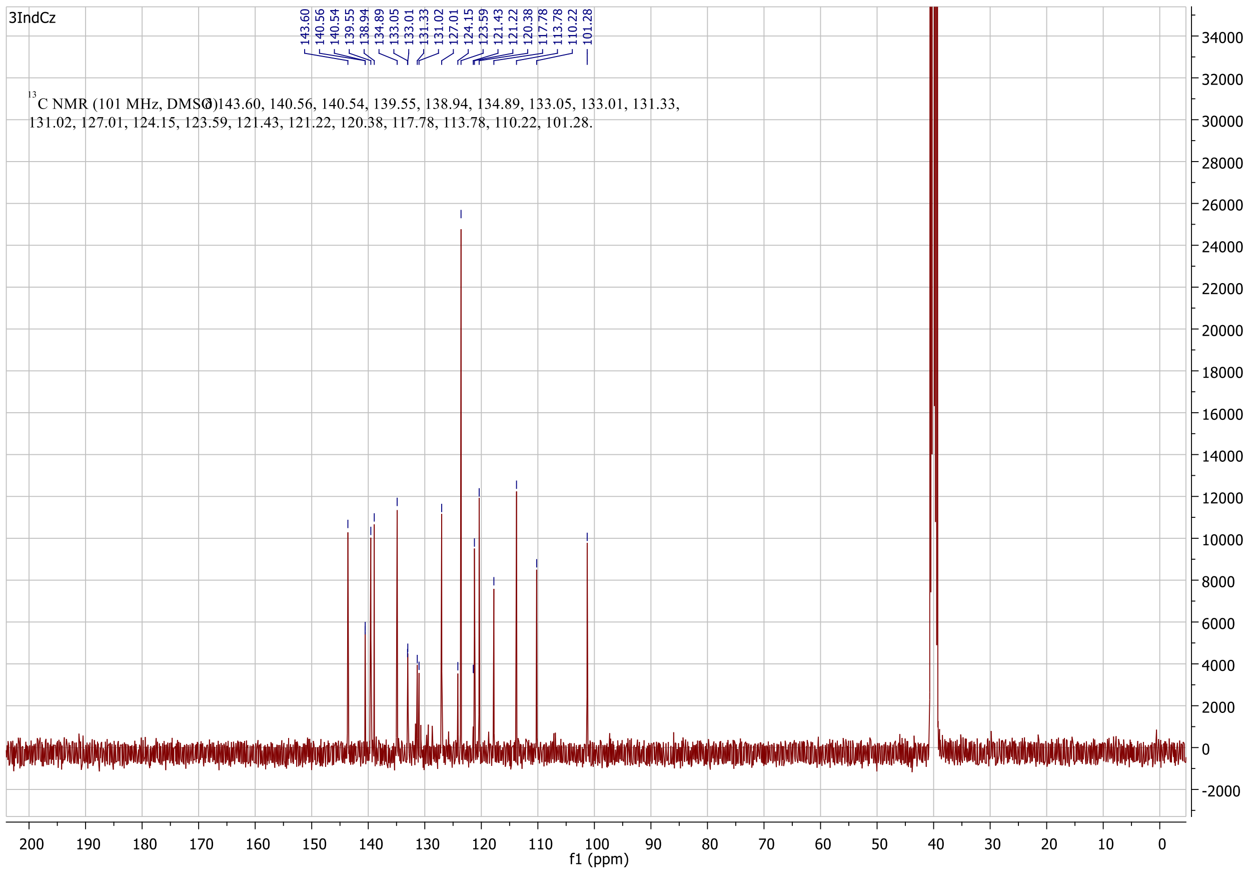

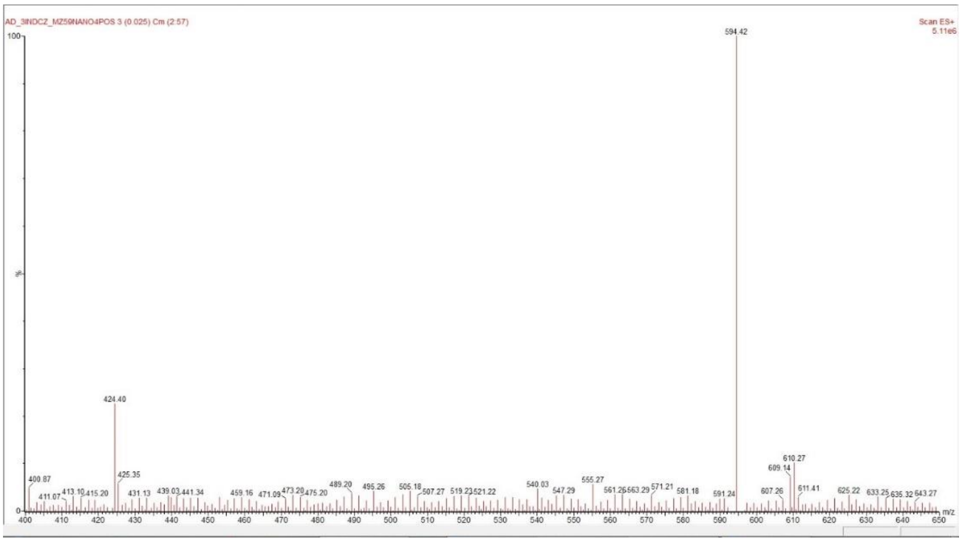

Figure S7. ^{1}H, ^{13}C NMR and MS spectra of 3IndCz.

References.

[S1] E. Skuodis, O. Bezvikonnyi, A. Tomkeviciene, D. Volyniuk, V. Mimaite, A. Lazauskas, A. Bucinskas, R. Keruckiene, G. Sini and J. V. Grazulevicius, *Org Electron*, 2018, **63**, 29–40.
